# Personal identification using a cross-sectional hyperspectral image of a hand

**DOI:** 10.1117/1.JBO.30.2.023514

**Published:** 2024-12-16

**Authors:** Takashi Suzuki

**Affiliations:** Osaka Metropolitan University, Center for Health Science Innovation, Smart Life Science Lab., Osaka, Japan

**Keywords:** hyperspectral imaging, biometrics, personal identification, artificial intelligence, MediaPipe Hands

## Abstract

**Significance:**

I explore hyperspectral imaging, a rapid and noninvasive technique with significant potential in biometrics and medical diagnosis. Personal identification was performed using cross-sectional hyperspectral images of palms, offering a simpler and more robust method than conventional vascular pattern identification methods.

**Aim:**

I aim to demonstrate the potential of local cross-sectional hyperspectral palm images to identify individuals with high accuracy.

**Approach:**

Hyperspectral imaging of palms, artificial intelligence (AI)-based region of interest (ROI) detection, feature vector extraction, and dimensionality reduction were utilized to validate personal identification accuracy using the area under the curve (AUC) and equal error rate (EER).

**Results:**

The feature vectors extracted by the proposed method demonstrated higher intra-cluster similarity when the clustering data were reduced through uniform manifold approximation and projection compared with principal component analysis and t-distributed stochastic neighbor embedding. A maximum AUC of 0.98 and an EER of 0.04% were observed.

**Conclusions:**

I proposed a biometric method using cross-sectional hyperspectral imaging of human palms. The procedure includes AI-based ROI detection, feature extraction, dimension reduction, and intra- and inter-subject matching using Euclidean distances as a discriminant function. The proposed method has the potential to identify individuals with high accuracy.

## 1 Introduction

Biometrics is an authentication method that utilizes physical characteristics unique to each individual to verify identity.^1^ Traditional biometrics, such as fingerprint,^2^ face,^3^ and palmprint,^4^ are commonly employed for personal authentication or identification purposes. However, many of these features are externally visible, rendering them less secure and more susceptible to counterfeiting. In contrast, internal biometrics provide increased security as they are not readily exposed and are difficult to replicate. Features such as vein patterns, inherent in the human body, offer enhanced security compared with external features as they cannot be captured from a distance.^5^^–^^7^

In recent times, the importance of identity verification has surged, particularly in areas such as offline payment processing and event access control. Biometric authentication has gained traction as a password-free solution due to the challenges associated with managing IDs and passwords. These methods are particularly resilient against identity theft due to their reliance on unique biological traits. Palm vein authentication, in particular, is renowned for its high accuracy and resistance to fraud as it relies on internal bodily information.^6^^,^^8^^,^^9^ This is attributed to the lower melanin deposits and fewer melanocytes in the skin tissues of the palm compared with other parts of the body, making it suitable for optical imaging.

Optical imaging techniques for biometrics have utilized optical coherence tomography (OCT)^10^^,^^11^ and photoacoustic tomography (PAT)^12^^,^^13^ for finger measurements. These modalities offer the capability to extend tissue measurements from 2D to 3D. OCT provides cross-sectional images of the fingertip, revealing features such as sweat gland distribution and papillary junctions in the epidermis layer. On the other hand, PAT utilizes 3D vein structures for biometric identification. Both approaches have demonstrated high authentication accuracy, suggesting that leveraging subcutaneous information for personal identification could enhance robustness against spoofing.

Hyperspectral imaging, a type of optical imaging, captures a sequence of images from the same scene across a broad spectrum of contiguous wavelengths.^14^ A hyperspectral image can be visualized as a hypercube structured in three dimensions, where the initial two dimensions represent the spatial geometry of the image (x,y) and the third dimension corresponds to the spectral wavelength (λ).^15^ Different light wavelengths penetrate various skin layers and illuminate different spectra.^16^^,^^17^ Therefore, the visible spectrum and near-infrared spectrum could enhance different layers of the palm and contain the most useful features for palmprint verification. Particularly, high spectral resolution reveals distinct vein patterns.^18^ Spectral information from hyperspectral imaging enables the detection of subcutaneous tissue structures,^19^ which vary significantly from person to person. In addition to biometrics by OCT and PAT, utilizing depth information from spectral data obtained through hyperspectral imaging can also be a potent tool for personal identification.

Hyperspectral imaging offers a wealth of information, yet processing it is complicated by the high dimensionality of the data space. In image processing, the size of an image significantly impacts the computational costs of various algorithms and operations. Larger images necessitate more memory, processing power, and time for tasks such as feature extraction, filtering, and recognition. In addition, reducing the required palm area decreases device cost and size.^20^ On the other hand, a cross-sectional image of a hyperspectral data cube comprises a continuous sequence of spectra,^14^ depicting texture patterns that may vary among individuals. By generating a cross-sectional image of a part of the palm, image size can be reduced while preserving all wavelength information along the cut line. Therefore, personal identification using a cross-sectional hyperspectral image is expected to reduce computational costs.

In generating a cross-sectional image of a hyperspectral image, region of interest (ROI) extraction is a crucial step as it directly impacts subsequent feature extraction and matching. Researchers are continuously exploring innovative methods to improve the accuracy and efficiency of hand-palm image registration for ROI extraction.^8^ Recent advancements in deep learning and computer vision have propelled the development of hand-pose recognition. Thus, an artificial intelligence (AI)-based ROI detection technique was employed to determine the cutting plane of a hyperspectral cube, automating, and simplifying the ROI setting process.

A hyperspectral imaging-based personal identification system proves to be a valuable tool, particularly in clinical settings where biometric identification can significantly reduce errors stemming from patient misidentification.^21^ Furthermore, hyperspectral imaging exhibits considerable potential in clinical applications, particularly in disease diagnosis and image-guided surgery.^22^ Therefore, integrating hyperspectral authentication with clinical hyperspectral devices is expected to enhance their usability.

In this study, personal identification was assessed using the proposed method on a self-built database. The aim of this study is to illustrate that a local cross-sectional hyperspectral palm image, retaining rich spectral information within a section, can accurately identify individuals. In addition, the efficacy and precision of region extraction using AI-based ROI settings were validated.

## 2 Methods

### 2.1 Experiment Setup

#### 2.1.1 Hyperspectral imaging system

**Fig. 1:**
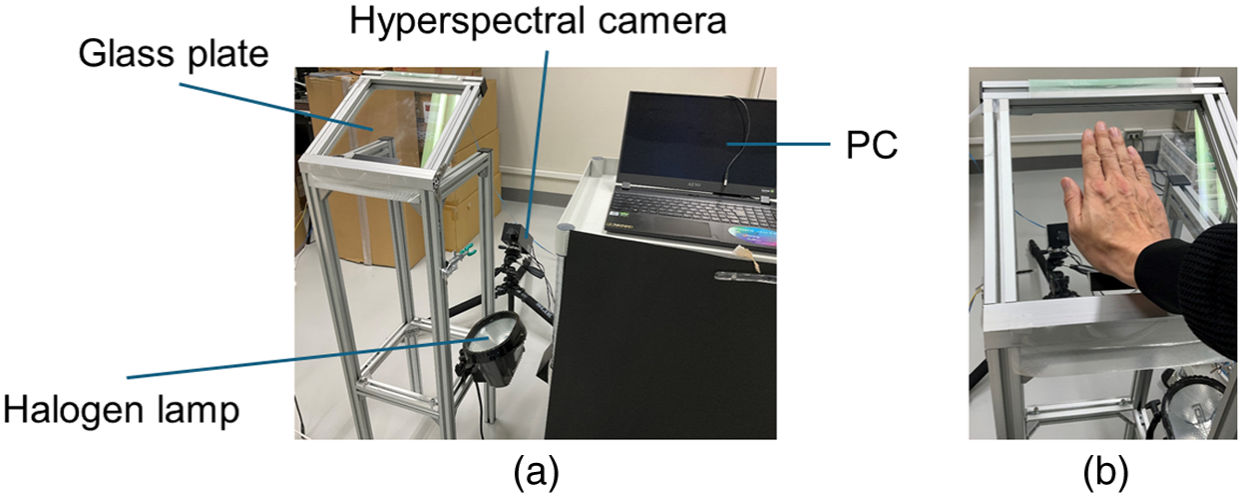
Experimental setup of hyperspectral imaging for acquiring palm data. (a) Hyperspectral imaging system consists of a hyperspectral camera, light source, and scanning section. (b) Example of palm scanning.

The experimental setup comprised a hyperspectral camera, a palm scanner, a broadband illumination light source, and a personal computer (Fig. 1). The hyperspectral imaging for the hand was established using a hyperspectral camera (NH-A-S, EBA, Japan, Japan), equipped with a single focus lens (f=12  mm, M118FM12, Tamron, Japan), providing a spectral resolution of 5 nm across the total range of 400 to 1000 nm [Fig. 1(a)]. The scanner featured a 240×240-mm scanning area, positioned 900 mm above the floor and tilted at an angle of ∼30  deg to the horizontal ground, with a 5-mm-thick high-transparency glass plate (OOKABE GLASS, Japan) inserted to transmit visible to near-infrared light [Fig. 1(a)]. Subjects were placed one palm on the glass plate to acquire hyperspectral images while maintaining the distance between the lens and the objects being photographed [Fig. 1(b)]. Palm placement on the scanner for measurements was unrestricted, except when the fingers were pointed forward. Hyperspectral images of the palm were captured through the glass using a low-angle shot from the hyperspectral camera. Positioned ∼500  mm behind the scanner, the hyperspectral camera’s lens tip was set ∼450  mm above the floor and tilted at an angle of ∼40  deg to the horizontal ground. A 500-W halogen lamp (CTW-1550, SANKYO CORPORATION, Japan), placed beneath the scanning section, illuminated the subject’s palm from the reverse side of the glass [Fig. 1(a)]. The lamp’s radiation raised the surface temperature of the glass by ∼3°C in 30 s. Camera control and data acquisition were managed using the manufacturer-provided software (NH Capture, EBA Japan, Japan). Spectral reflection was captured using a hyperspectral camera, and the hyperspectral cube data (640×480  px and 121 bands) were stored on the personal computer’s hard disk. The scan rate was set at 20  lines/s, and the camera exposure time was set at 0.05 s (50 ms). The total scan time was ∼24  s. The lateral resolution (sensor direction) was 0.36  mm/px, whereas the axial resolution (scan direction) was 0.42  mm/px.

#### 2.1.2 Image processing

The original hyperspectral image was calibrated for white and dark balance using the following equation:^23^

$$I_{\mathrm{ref}}=\frac{I_{\text{raw}}-I_{\text{dark}}}{I_{\text{white}}-I_{\text{dar}}}.$$ (1)

In the provided equation, $I_{\mathrm{ref}}$ represents the relative reflectance of the hyperspectral image, scaled from no reflectance at a value of 0 to 100% reflectance at a value of 1. $I_{\text{raw}}$ denotes the original image data, whereas $I_{\text{white}}$ and $I_{\text{dark}}$ stand for the white reflectance image data and the dark-current dark image data, respectively. The white reference image was obtained under the same conditions as the raw images using a white surface board (ColorChecker White Balance, X-Rite, Grand Rapids, Michigan, United States). The dark reference image was acquired by turning off the light source and fully covering the camera lens with its black cap. This calibration process was executed using ImageJ.^24^ The calibrated hyperspectral image was saved in a 32-bit tiff format.

#### 2.1.3 Region of interest detection for image registration

**Fig. 2:**
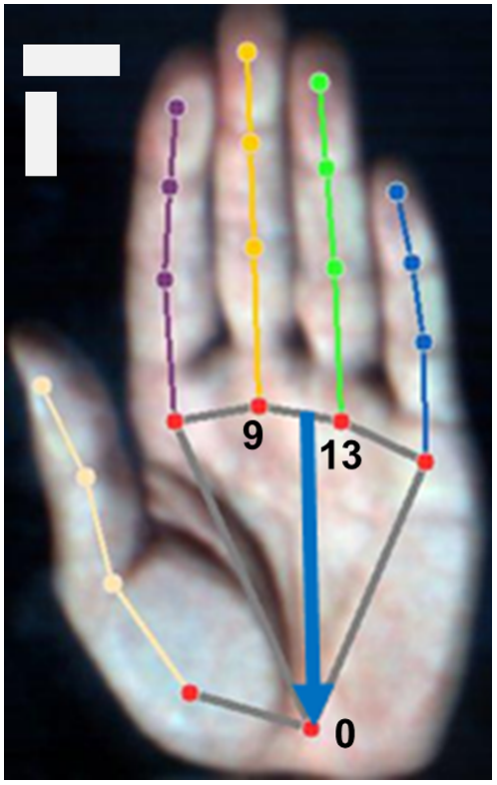
Hand landmarks model of MediaPipe. Blue arrow indicates a line ROI. Horizontal and vertical scale bars indicate 20 mm.

To ensure consistency in the region extracted from the palm for each measurement, landmarks were detected on a palm image using MediaPipe Hands (version 0.10.1), an open-source image processing machine learning library developed by Google Inc.^25^^–^^27^ The development environment consisted of a Jupyter notebook (version 6.5.4) with the Python programming language (version 3.11.5). To enhance the precision of recognition by MediaPipe Hands, a pseudo-RGB image was generated from the hyperspectral image, following a previous study.^28^ RGB images were produced using in-house LabVIEW software (LabVIEW 2020, National Instruments, Austin, Texas, United States). Subsequently, Google’s pre-trained MediaPipe Hand landmarks model was applied to the RGB hand palm image, automatically generating 20 landmarks on the image (Fig. 2). These landmarks were used to draw a straight line as an ROI through landmark #0, and the midpoint between landmarks #9 and #13 was determined using ImageJ (version 1.53t). The average length of the line ROI placed on the image was 76.19 mm. Further details regarding all measurement values for each subject are listed in Table S1 in the Supplementary Material. Prior to tracing the ROI onto a hyperspectral image, a Gaussian filter (σ=2) was applied to both the spatial and spectral directions for image noise reduction using ImageJ. During the preliminary test, it was observed that larger σ values could lead to numerical overflow issues, resulting in pixel values reaching infinity. To avoid this problem and ensure stable and accurate filtering, it was determined that σ=2 provided an optimal balance. This value could effectively reduce noise while maintaining the integrity of important image features without causing numerical overflow.

#### 2.1.4 Feature extraction

**Fig. 3:**
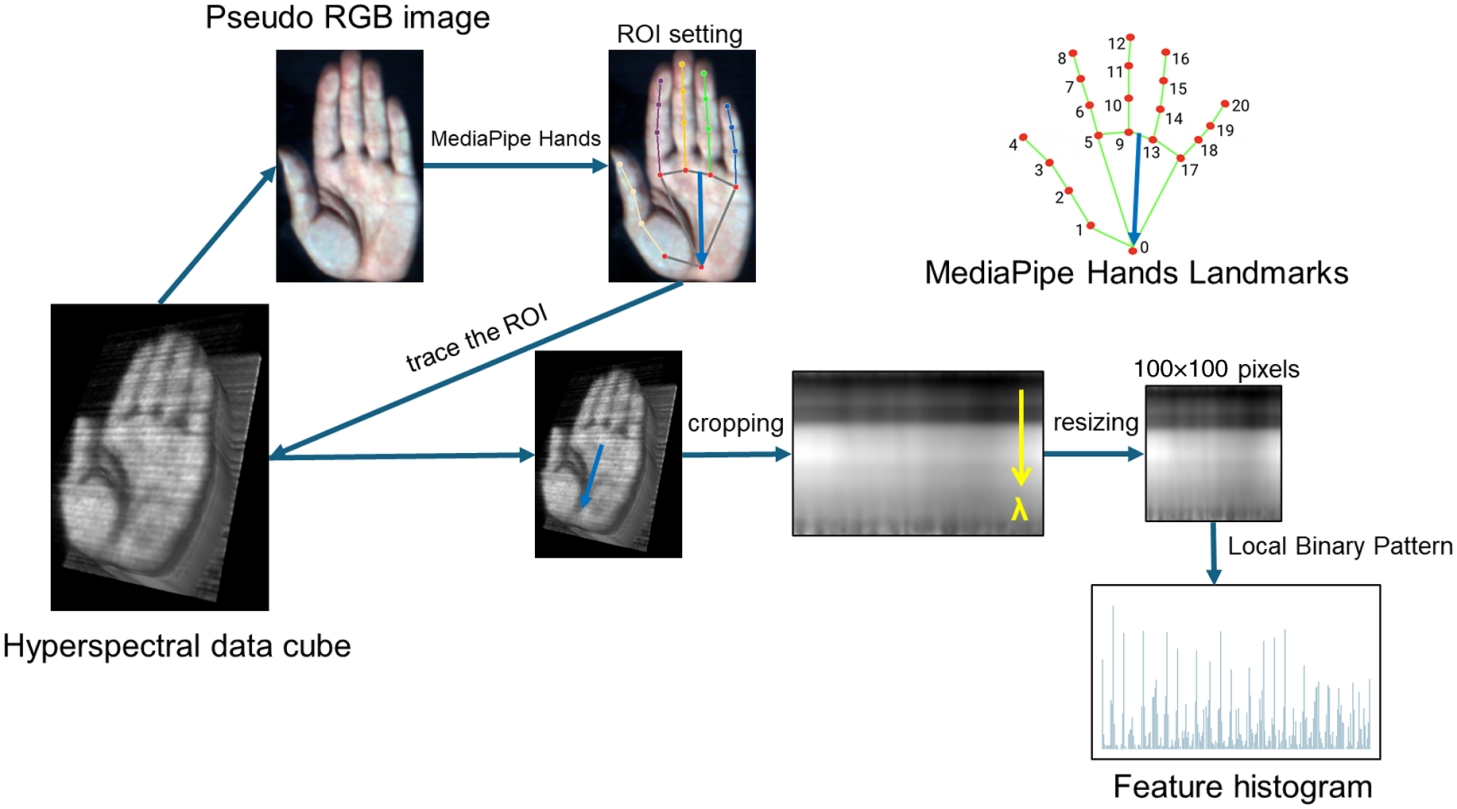
Visualization of feature vector extraction.

Figure 3 depicts a schematic of the imaging procedure employed for feature extraction. Following the ROI detection, the hyperspectral image was resliced along the straight line ROI using the “Reslice” function of ImageJ, resulting in a 2D spatial-spectral image. The flowchart in Fig. 3 outlines the feature extraction process. Subsequently, the resliced image was resized to 100×100  px via bilinear interpolation using a resize plug-in for ImageJ software to standardize the image size. This resized image was subsequently converted to 8-bit grayscale and saved in JPEG format.

The local binary pattern (LBP)^29^ was utilized to extract features from the resized 2D spatial-spectral images. The resized image was divided into 25 non-overlapping square sub-regions of the same size (20×20). Features were extracted from each sub-region, and histograms were constructed for each region. These histograms were concatenated into a long vector, serving as the feature vector for the hand hyperspectral image. Image processing was conducted using the IMAQ Extract LBP Feature Vector VI^30^ of LabVIEW. Each histogram comprised nine bins, resulting in a feature vector with dimensions of 225 (5×5×9).

#### 2.1.5 Clustering

The feature values extracted from the biometrics underwent analysis using K-means with principal component analysis (PCA),^31^ t-distributed stochastic neighbor embedding (t-SNE),^32^ and uniform manifold approximation and projection (UMAP),^33^ implemented using the Python package. Using Python’s scikit-learn Version 1.3, these three dimensionality reduction algorithms were executed with n_components = 2 and random_state = 0. For PCA, the explained variance ratio was [0.4215, 0,10992], which accounted for only 53.1% of the variance. Although setting n_components = 8 exceeded 80% variance, the explained variance rations for PC3 to PC8 were significantly lower. Therefore, selecting PC1 and PC2 appeared reasonable. Even with n_components = 8, no combination of components except for PC1 and PC2 improved clustering accuracy. t-SNE and UMAP were executed with the n_components = 2 and random_state = 0, which was consistent with that of the PCA. This ensured dimensionality reduction consistency, facilitated comparison, simplified visualization, and guaranteed reproducibility. In addition, the PCA results with n_components = 2 aligned with the k-means (k=10) clustering results. Similarly, the results from t-SNE and UMAP also matched the k-means results. Therefore, in this study, setting n_components = 2 was adopted for each clustering process. These dimensionality reduction techniques transform the feature vector from a high-dimensional space to a low-dimensional space (from 225 to two dimensions in this study), retaining some meaningful properties of the original data.

The similarity among different biometric feature vectors after dimension reduction was evaluated using Euclidean distance. A small Euclidean distance is expected between intra-subjects, whereas the distance between inter-subjects is larger. In addition, the statistical significance of the difference between the mean Euclidean distances of the two populations (intra-subject and inter-subject) was assessed by an unpaired Welch’s t-test using Kaleidagraph 5.0 (HULINKS Inc., Japan).

To define the reference threshold value, the false acceptance rate (FAR) and false rejection rate (FRR) were calculated. FAR is defined as the number of incorrectly accepted individuals divided by the total number of incorrect matches, whereas FRR is defined as the number of incorrectly rejected individuals divided by the total number of correct matches. There were a total of 900 intra-class and 9000 inter-class matches. FAR and FRR were calculated at each threshold of the Euclidean distance, increasing in steps. This calculation was performed using an in-house LabVIEW code (LabVIEW 2020, National Instruments, Austin, Texas, United States). In addition, recognition performance was assessed using the equal error rate (EER), which is the point where FAR and FRR are equal.

The clustering performance for user identification was evaluated using receiver operating characteristics (ROC) curves, plotting the true acceptance rate (TAR), defined as 1 − FRR, as a function of FAR. To quantitatively assess the performance based on ROC curves, the area under the curve (AUC) was computed using scikit-learn, an open-source Python library.

#### 2.1.6 Image processing times

The image processing times after hyperspectral image acquisition from filtering to clustering were evaluated. A laptop computer consisting of an Intel^®^ Core™ i7-1068NG7 central processing unit (CPU) and 32 GB of random access memory was used for image processing. Image processing in this study was not fully automated; however, different software packages such as Jupyter Notebook (for annotation and clustering), LabVIEW (for creating RGB images and feature extraction), and ImageJ (for denoising and transforming images) were used for various steps. The execution time for each cell was measured using the “%%time” magic command in Jupyter Notebook. The total processing time was calculated by summing the wall times as the execution times of all cells. The processing time in LabVIEW was measured by combining the tick count function with a sequence structure. The execution time was determined by calculating the difference between initial and final tick counts. The processing time in ImageJ was recorded as the time displayed on the user interface.

### 2.2 Subjects

In this study, 10 healthy adults (seven males and three females) participated. The age range of the participants was from 24 to 47 years old. Each subject had their palm scanned 10 times using the imaging system. One subject was measured five times on two separate days, whereas another subject was measured five times at different times on the same day. The other subjects were measured 10 times continuously with a short break between each measurement. To protect their eyes from the illumination light, the subjects wore safety glasses (LG2, Thorlabs, Newton, New Jersey, United States).

### 2.3 Ethic Statement

This project was approved by the Ethics Committee of the Center for Health Science Innovation at Osaka City University (approval No. 42, June 30, 2021). All subjects signed an informed consent form before enrollment in the study.

### 2.4 Additional Experiments

Additional experiments were conducted to examine the effects of hand placement and light source position and intensity on imaging and personal identification results. These experiments are detailed in Sec. S3 in the Supplementary Material and include Figs. S4 and S7 in the Supplementary Material related to the methodology, which can be referred to for further information.

## 3 Results

**Fig. 4:**
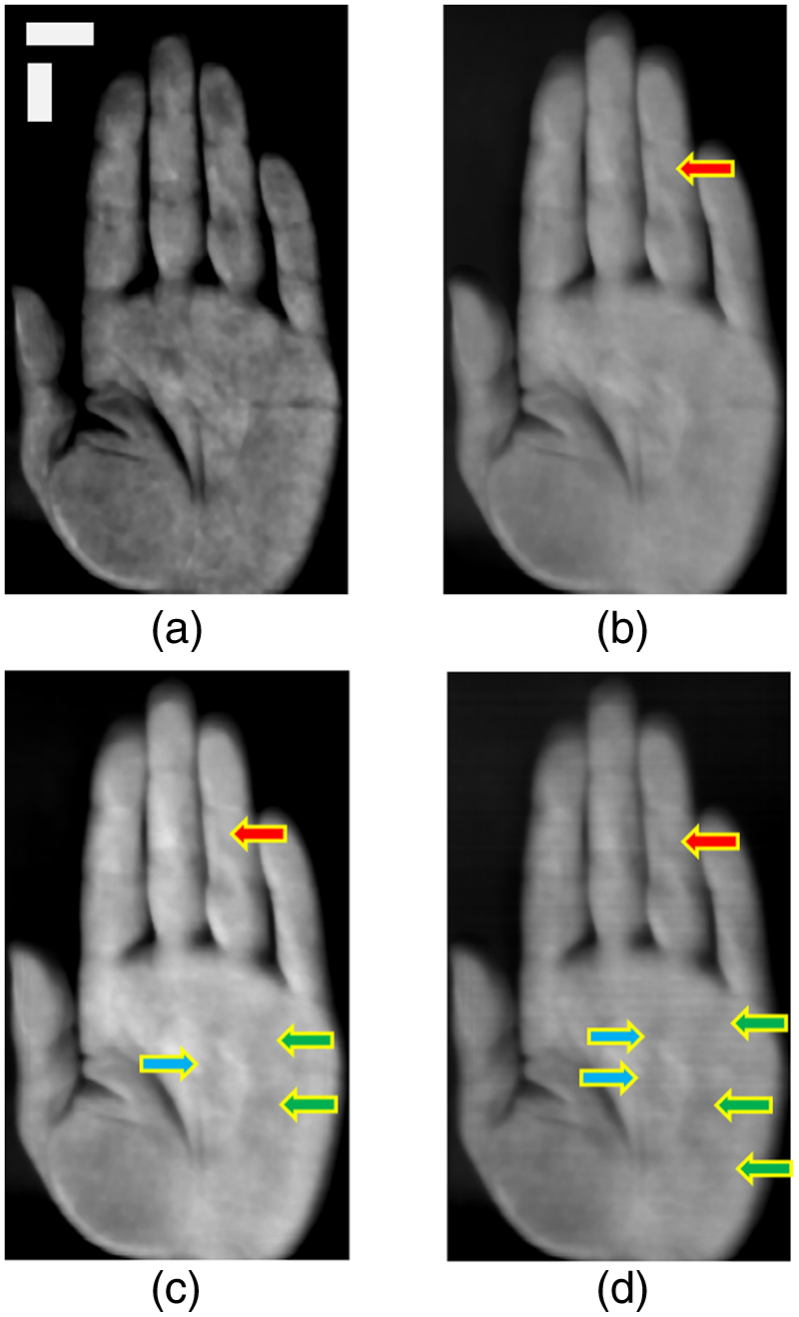
Averaged image of 20 spectral images every 5 nm. (a) Averaged from 500 to 600 nm. (b) Averaged from 600 to 700 nm. (c) Averaged from 700 to 800 nm. (d) Averaged from 800 to 900 nm. The arrows indicate vein-like patterns. Each color represents a different pattern, and the same color indicates that the vein-like patterns are continuous. Horizontal and vertical scale bars in the top-left image indicate 20 mm. These data are from subject 1.

The representative results of the hyperspectral hand images averaged over a certain wavelength band are shown in Fig. 4. All spectral images from the same subject are presented in Fig. S1 in the Supplementary Material. In a low waveband, a patchy pattern was observed [Fig. 4(a)]. As the waveband lengthened, the patchy pattern disappeared, and vein-like patterns were observed [Figs. 4(b)–4(d)].

**Fig. 5:**
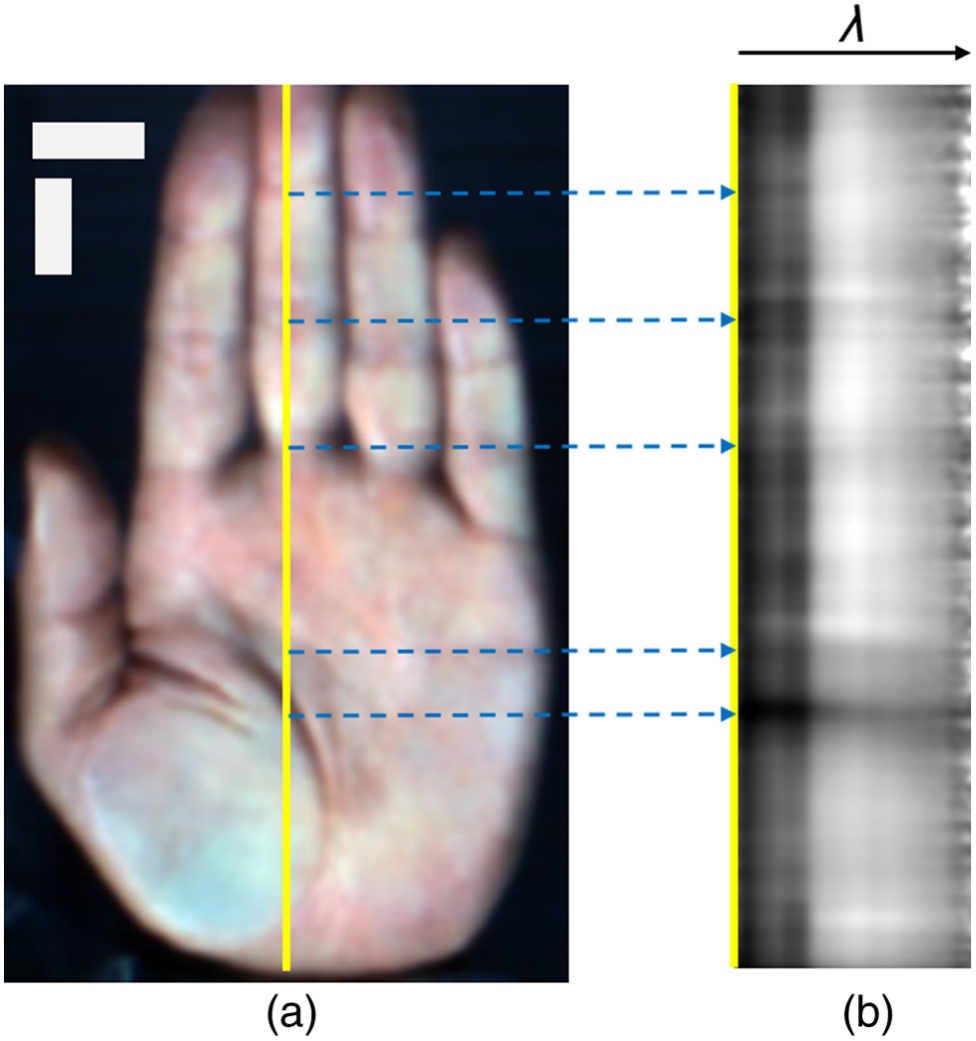
Correspondence between a palm and a cross-sectional image. (a) A pseudo-color image reconstructed from a hyperspectral image. The yellow line indicates a line ROI. (b) Cross-sectional image. Blue dot arrows indicate the same point between panels (a) and (b). The black arrow indicates the wavelength (λ) direction. Horizontal and vertical scale bars in the left image indicate 20 mm.

Figure 5 shows an example of a cross-sectional hyperspectral image along a line of interest. The cross-sectional image consisted of a series of spectra, ranging from 400 to 1000 nm in 5-nm increments. The sequence of the spectra depicted a textured pattern. In addition, shadow lines perpendicular to the cutting line were observed in the images. These shadow lines corresponded to the palm surface morphology, such as the lines of interphalangeal joints, palm lines, palmprints, and hand wrinkles [Fig. 5(a)].

**Fig. 6:**
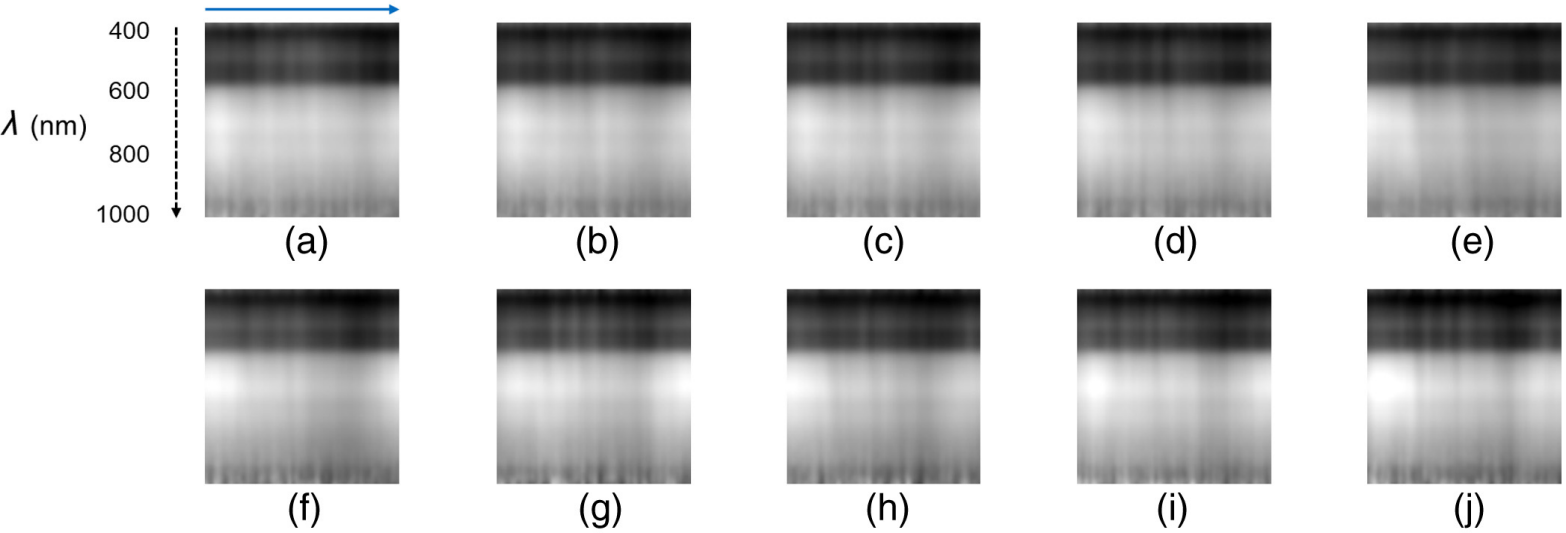
Image-processed cross-sectional hyperspectral images of subject 1. A black dashed vertical arrow from the top to bottom indicates the wavelength (λ) direction. The blue horizontal arrow from left to right corresponds to a line ROI in Fig. 2. (a) First measurement result. (b) Second measurement result. (c) Third measurement result. (d) Fourth measurement result. (e) Fifth measurement result. (f) Sixth measurement result. (g) Seventh measurement result. (h) Eight measurement results. (i) Ninth measurement result. (j) Tenth measurement result. Each image size is 100×100  px.

**Fig. 7:**
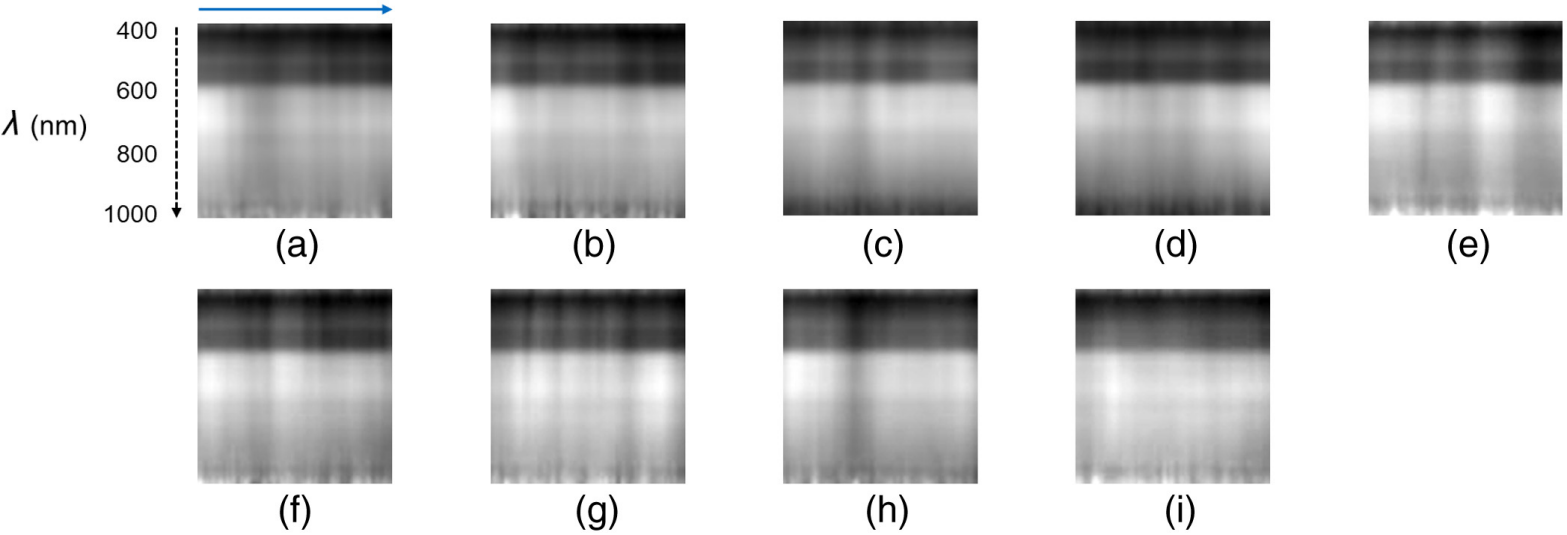
Image-processed cross-sectional hyperspectral images of hands from different subjects. The black dashed vertical arrow from top to bottom indicates the wavelength (λ) direction. The blue vertical arrow from left to right corresponds to a line ROI in Fig. 2. (a) Representative result of subject 2. (b) Representative result of subject 3. (c) Representative result of subject 4. (d) Representative result of subject 5. (e) Representative result of subject 6. (f) Representative result of subject 7. (g) Representative result of subject 8. (h) Representative result of subject 9. (i) Representative result of subject 10. Each image size is 100×100  px.

The processed cross-sectional images of one subject (Fig. 6) and different subjects (Fig. 7) are shown. Overall, the cross-sectional hyperspectral image exhibits a layered structure caused by the luminance gradient. The brightness was darker in the short-wavelength range and brighter in the middle-to-long wavelength range, with distinct brightness distributions observed in each layer. In addition, the cross-sectional images contained vertical shadow lines. Images from the same subject showed a similar pattern, whereas images from different subjects tended to display different patterns. The feature vectors extracted by the LBP histogram from the data in Figs. 6 and 7 are shown in Figs. S2 and S3 in the Supplementary Material, respectively. Similar trends are observed for the feature vectors of a single subject, whereas the histogram patterns from different subjects did not show similarities.

**Fig. 8:**
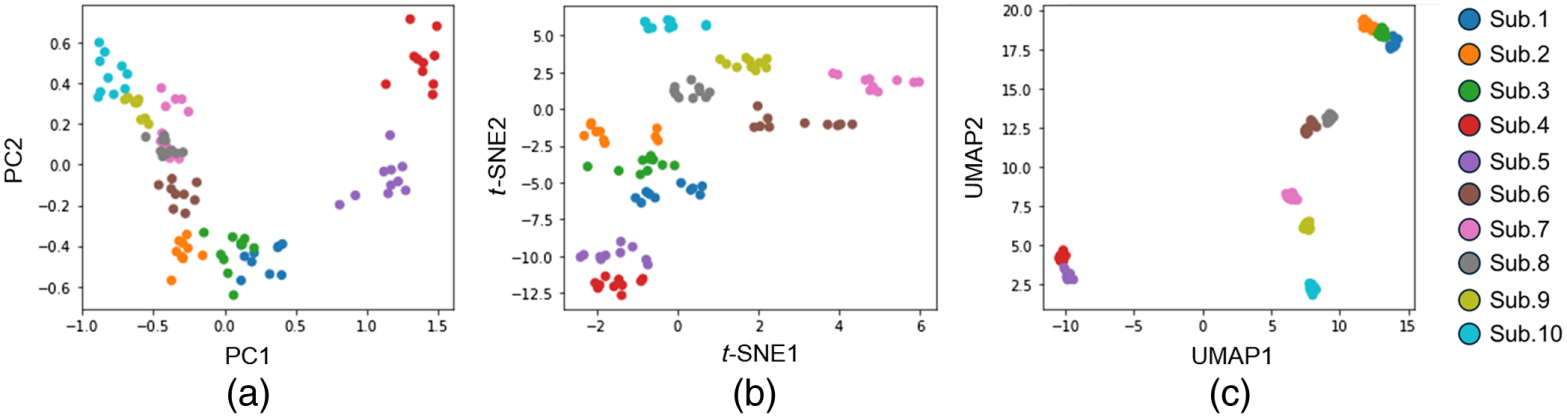
Comparison of different dimensional reduction algorithms on the self-built database. Each color indicates data from the same subject. (a) Feature size is reduced to dimension 2 by PCA. PCA plot shows two principal components (PC1 and PC2). (b) Feature size is reduced to dimension 2 by t-SNE. t-SNE plot illustrates 2D embedding (t-SNE1 and t-SNE2) of the dataset. (c) Feature size is reduced to dimension 2 by UMAP. UMAP plot depicts the 2D embedding (UMAP1 and UMAP2) of the dataset.

Figure 8 shows the performance of K-means with PCA (a), t-SNE (b), and UMAP (c) clustering of the feature vectors extracted from the cross-sectional image using LBP. For each case, the data were reduced to two dimensions, and the plots were colored based on the ground truth of the self-built data. The results visually illustrate that the data clusters are well separated. Notably, UMAP shows the best clustering, t-SNE presents better clustering than PCA, and PCA also shows good clusters.

**Fig. 9:**
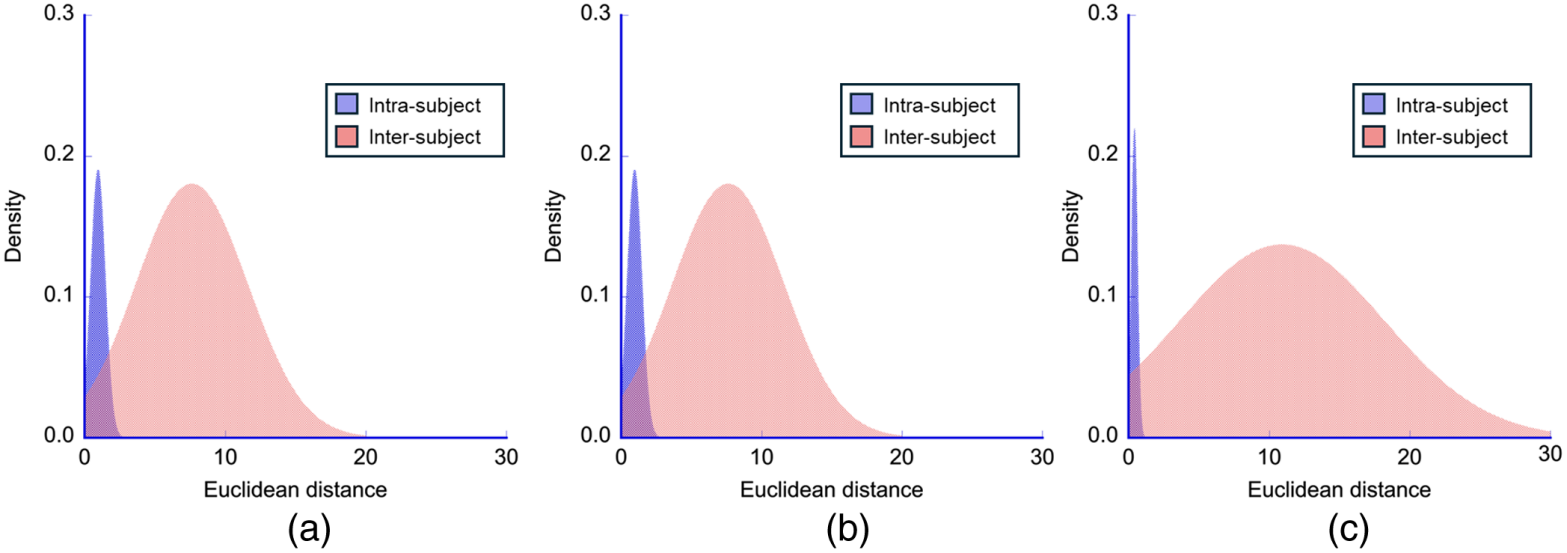
Probability distribution histogram. (a) Feature size reduced to dimension 2 by PCA. (b) Feature size reduced to dimension 2 by t-SNE. (c) Feature size reduced to dimension 2 by UMAP. Blue fill represents the intra-subject distribution, whereas light pink fill represents the inter-subject distribution.

**Fig. 10:**
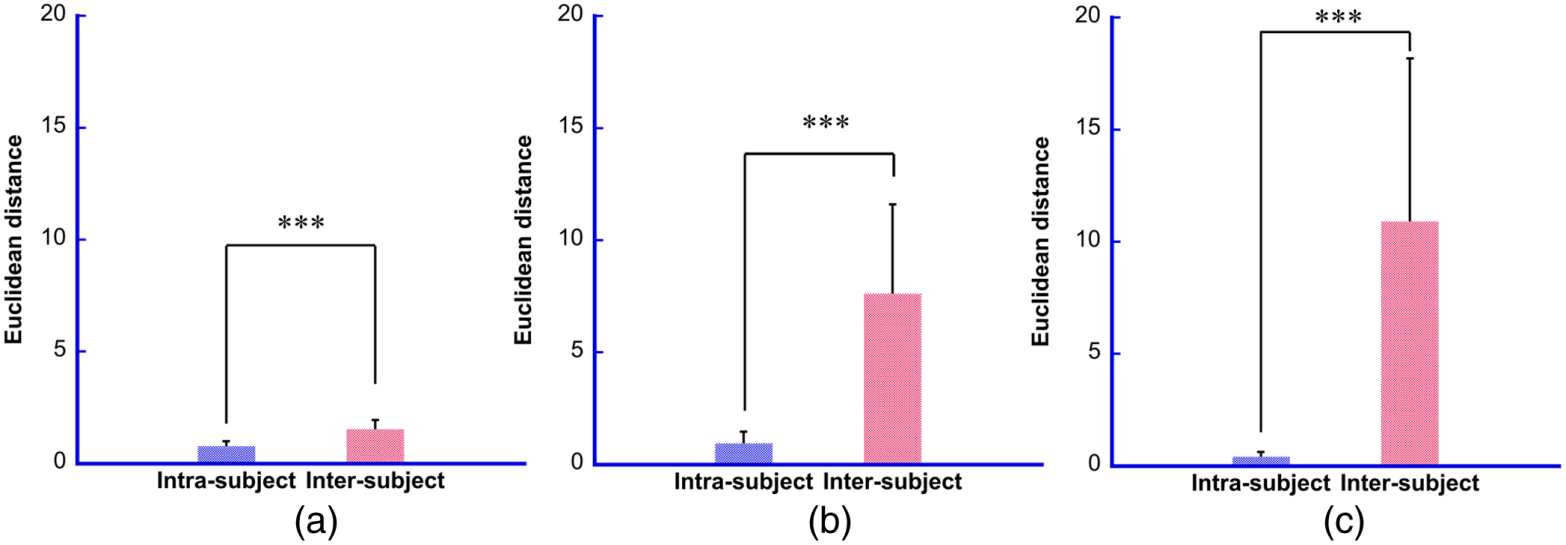
Significant differences of intra- and inter-subject matching. (a) Feature size reduced to dimension 2 by PCA. (b) Feature size reduced to dimension 2 by t-SNE. (c) Feature size reduced to dimension 2 by UMAP. Values are means ± standard deviations. ***P<0.0001. The blue bar represents intra-subject data, whereas the light pink bar represents inter-subject data.

To determine the clustering performance of the biometrics with hyperspectral imaging, the distributions of the Euclidean distance-based discriminant function were computed. The Euclidean distances of inter- and intra-subject matching were analyzed (Fig. 9). In all cases, the distribution curves with a Gaussian fit exhibited an obvious bimodal shape. It can also be observed that inter-subject distances are described by a wide distribution as opposed to intra-subject distances, which are captured by a remarkably peaked distribution. Furthermore, the inter-subject distances were significantly larger than the intra-subject distances in all cases (P<0.0001) (Fig. 10).

Table 1 summarizes the mean, standard deviation, maximum, and minimum values derived from Figs. 9 and 10.

**Table 1:** Summary of mean, standard deviation, maximum, and minimum values derived from probability distribution and mean comparison.

	PCA		t-SNE		UMAP	
	Intra-subject	Inter-subject	Intra-subject	Inter-subject	Intra-subject	Inter-subject
Mean	0.77	1.52	0.94	7.62	0.41	10.91
Standard deviation	0.22	0.42	0.52	3.98	0.22	7.72
Maximum	1.39	2.59	2.68	18.88	1.30	23.25
Minimum	0.36	0.71	0.09	0.92	0.02	0.42

**Fig. 11:**
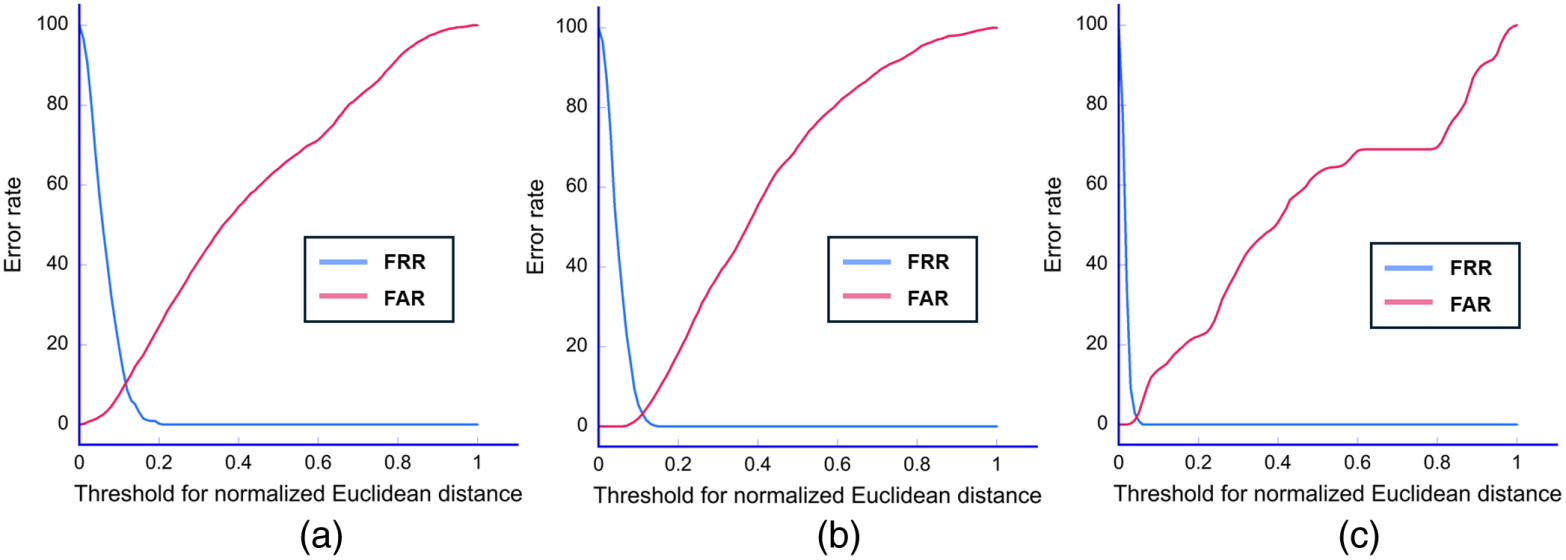
Changes of false acceptance rate (FAR, bright pink line) and false rejection rate (FRR, light blue line) under different thresholds. (a) Feature size reduced to dimension 2 by PCA. (b) Feature size reduced to dimension 2 by t-SNE. (c) Feature size reduced to dimension 2 by UMAP.

To assess authentication accuracy, FAR and FRR were used. Figure 11 illustrates the changes in FAR and FRR under different Euclidean distances of each dimensionally reduced space using PCA, t-SNE, and UMAP. The abscissas of these graphs represent the threshold for a normalized Euclidean distance within each dimensionally reduced space, ranging from 0 to 1. If the normalized Euclidean distance between the two data points in the space is closer to 0, this indicates a higher possibility of being the same subject. Conversely, a distance closer to 1 indicates a higher possibility of being a different subject. By setting a threshold within range, distances below the threshold were considered the same subject, whereas distances above the threshold were considered different subjects. Therefore, the increase in threshold value leads to a decrease in FRR, albeit an increase in FAR. Similarly, a decrease in threshold value leads to a decrease in FAR, albeit an increase in FRR. The threshold value can be found at the intersection of the FAR and FRR plots. The value at this intersection point represents the EER. The smallest threshold was observed for UMAP, followed by t-SNE and PCA.

**Fig. 12:**
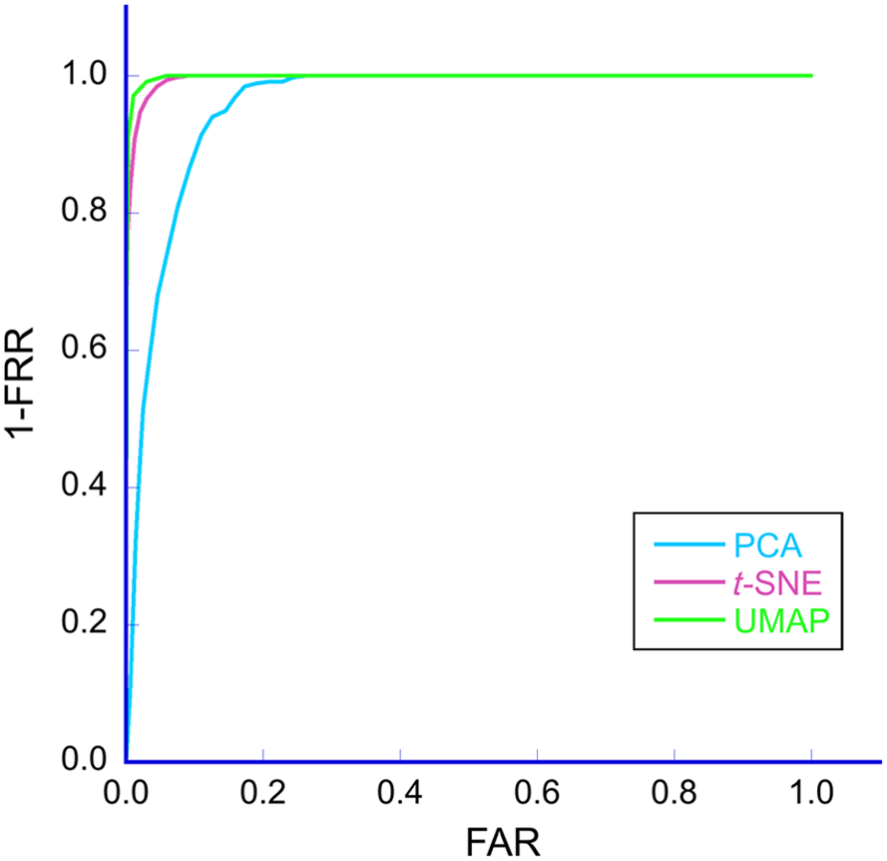
Receiver operating characteristic (ROC) curves of three different dimension reduction algorithms.

As shown in Fig. 12, the ROC curve was calculated. The AUC was used as the optimization objective because it provides a good representation of ROC performance. The verification results for the EER and AUC are reported in Table 2. It can be observed from Table 2 that UMAP demonstrated better performance than the other methods. Therefore, additional experiments investigating the effects of palm and light source positions and light source intensity were clustered using only UMAP. These results are shown in Figs. S6, S9, and S11 in Sec. S3 of the Supplementary Material. In addition, the variabilities in clustering using UMAP in the additional experiments are shown in Table S2 in Sec. S4 of the Supplementary Material.

**Table 2:** Verification performance by different methods.

Algorithm	EER (%)	AUC (%)
PCA	0.11	88.61
t-SNE	0.11	96.79
UMAP	0.04	98.02

The hyperspectral imaging process required 24 s; however, using two light sources reduced this to 8 s (see the Supplementary Material for details). For image processing, the 3D Gaussian filter noise reduction required 223.8 ms, conversion of the hyperspectral image to an RGB image required 4935.8 ms, MediaPipe Hands annotation required 29.9 ms, ROI setup and cross-sectional image extraction required 81.2 ms, and LBP feature vector extraction required 0.6 ms. Finally, the total image processing time was 5271.3 ms (∼5.3  s).

## 4 Discussion

In this study, an effective biometric technique using cross-sectional hyperspectral imaging of the palm was proposed. The cross-sectional hyperspectral image represented a pattern inherent to each person. The developed system uses MediaPipe Hands, a machine learning library, to automatically set the ROI without complex image registration. Despite the ROI being smaller compared with the entire palm, the optimal performance achieved an area of 0.98 under the ROC curve, with an EER of 0.04% at its highest performance. This technique will be incredibly beneficial because it has demonstrated high accuracy in personal authentication, leading to the development of a more secure bio-hyperspectral imaging system.

Personal verification and identification using palm images have drawn considerable attention.^34^ The palm vein pattern, a dense network of veins spanning the entire palm, is also used for authentication. However, a large palm scanning area increases data acquisition and analysis time. Nayar and Tomas^20^ proposed using a partial palm vein pattern for authentication, which has sufficient identification performance. They also stated that this technique will help reduce the size of the device. In this study, the palm scan area was reduced to a single line of the hyperspectral image. Although spatial information on the palm vein distribution was lost, the cross-sectional hyperspectral image of the palm was extremely informative in the wavelength direction. The hyperspectral images of the palm provided different features at each wavelength.^17^^,^^35^ These features were generated by the distribution of absorption in the skin and the different tissue penetration depths of light wavelengths.^16^ Patchy patterns were observed in the short waveband [Fig. 4(a)], as reported by Sato et al.^18^ The cross-sectional hyperspectral image displayed a gradient pattern with variations layering from the short- to long-wavelength direction. The gradient pattern in the wavelength direction corresponded to the luminance change in the hyperspectral image (Fig. S1 in the Supplementary Material) across all spectral bands. The low-brightness layer, corresponding to ∼400 to 600 nm, represented the patchy pattern of the spectral image appearing as darker areas (Figs. 6 and 7). Moreover, vein-like patterns were identified in some locations in the longer waveband. The high-brightness layer over 600 nm depicted another pattern as the vessel-like pattern of the spectral image also appeared as darker areas (Figs. 6 and 7). The lower brightness at shorter wavelengths in hyperspectral imaging can be attributed to two primary factors. First, the sensor sensitivity is lower at the spectral edges. Second, shorter wavelengths can be absorbed by the numerous capillaries near the skin surface. In contrast, the higher brightness at mid to long wavelengths is caused by deeper penetration, where fewer and larger blood vessels can be present compared with capillaries. Building on the second reason, in the hyperspectral image, patchy patterns were observed at shorter wavelengths, which disappeared as the wavelength increased, revealing vein-like patterns. The distribution of capillaries and veins in the skin differs from person to person. Personal identification using vascular patterns typically uses a 2D distribution; however, blood vessels are distributed in a 3D manner. Hyperspectral imaging uses different wavelength lights to penetrate the skin at different depths; therefore, cross-sectional hyperspectral images would reflect blood vessel distribution in the depth direction. Consequently, cross-sectional images could provide a unique pattern for each person to identify the individual. In addition, the palm lines matched with the striped shadows in the cross-sectional hyperspectral images (Fig. 5). The cross-sectional hyperspectral image includes information not only on the inside of the body but also on the surface. Multi-biometrics, such as palm print and finger knuckle print,^36^ palm print and dorsal hand veins,^37^ and palm print and palm veins,^38^ have succeeded in efficiently improving accuracy. Hence, it was suggested that cross-sectional images could have sufficient characteristics for personal identification, even within limited regions. The cross-sectional image at the same location would have an individual-specific pattern, thus enabling effective authentication.

Extracting the ROI is a critical and essential step in the palm recognition process, as the location of the ROI significantly impacts feature extraction within the palm image. Most ROI extraction algorithms utilize key points between fingers to establish a coordinate system. In contactless imaging, it is important to note that palm images present numerous translational and rotational variations. In this study, an AI-based approach, MediaPipe Hands, was used to extract the ROI from the palm images. Hand landmarking tools frequently produced incorrect landmarks on spectral monochromatic images. The hand pose estimation model is capable of predicting hand poses using only RGB input.^25^ Consequently, the reconstructed RGB images from the hyperspectral image showed accurate landmarks. The feature vector extracted using this ROI could classify each individual. Therefore, the ROI using MediaPipe Hands succeeded in selecting almost the same position each time, although effects may have occurred from filtering for noise reduction and interpolation in the resizing process. Alternatively, the hyperspectral cross-sectional images from nearby locations may not exhibit significant differences. The palm scan area in this study was not as large as the palm of the hand, which allowed the hand to be placed anywhere. However, the orientation and position of the hands are generally decided. In fact, in one subject (subject 1), some of the hand placements in the ten scans changed positions purposely; for example, the fingertips were positioned at angles ranging from ∼0 to 90 deg. However, these features from the subjects using the ROI detection were almost identical (Fig. 6). Therefore, the AI-based ROI placement technique is robust against misalignments and rotations. Thus, this technique may be superior to conventional image registrations.

The cross-sectional image extracted from the ROI was converted into a feature vector using LBP, which is widely used for face recognition.^39^ To visualize the relationships in the data, high-dimensional feature vectors were converted to two dimensions using dimensionality reduction algorithms such as PCA, t-SNE, and UMAP (Fig. 8). These results show 10 clusters of the 10-subject dataset based on their similarity. Consequently, the texture patterns in the cross-sectional hyperspectral images (Figs. 6 and 7) and the feature vectors (Figs. S2 and S3 in the Supplementary Material) contained individual features. The clustering results of the UMAP dimension reduction data revealed a high agglomeration of data for each label [Fig. 8(c)]. UMAP is a method where similar data in the original feature space are plotted closely after dimension reduction.^33^ In a comparison of the performance of dimensionality reduction techniques in clustering, UMAP provided better results than the other two algorithms.^40^ In addition, UMAP has a rigorous mathematical foundation; however, it is straightforward to utilize with a scikit-learn-compatible API.^41^ UMAP is also one of the fastest manifold learning implementations available and is significantly faster than most t-SNE implementations.^41^ Therefore, UMAP was considered advantageous not only in terms of clustering accuracy, albeit also in terms of processing speed.

To measure the effectiveness of the authentication system, the FRR and FAR were calculated using the Euclidean distance between the dimensionally reduced feature vectors as the discriminant function. The Euclidean distance for intra-subject comparisons was small, whereas that for the inter-subject comparisons was large. Across all dimensionality reduction procedures, the distribution of similarity scores for inter-subject comparisons showed high variability, indicated by a wide distribution. In contrast, intra-subject comparisons exhibited low variability, indicated by a sharply peaked distribution (Fig. 9 and Table 1). In addition, the mean Euclidean distance for inter-subject comparisons was significantly larger than for intra-subject comparisons (P<0.0001) for all algorithms (Fig. 10). The EER, which occurs when FRR equals FAR, is frequently used to provide a synthetic evaluation of a system’s detection capability. The smaller the EER, the better the model performance. Even herein, the smallest EER was observed for UMAP (Fig. 11 and Table 2).

The performance of authentication using different dimensionality reduction techniques was compared through ROC curves, which plot the FRR and TAR (1 − FRR) as a function of the FAR. The proposed method showed better recognition performance when the ROC curve was closer to the axis. To quantitatively evaluate the performances based on ROC curves, the AUC was calculated. The ROC curves and AUC indicated that UMAP demonstrated the best performance (Fig. 11 and Table 2). Thus, hyperspectral cross-sectional images can be effectively used for personal identification, with UMAP being one of the most suitable methods for dimensionality reduction.

Other personal identification techniques with optical imaging modalities, such as OCT^11^^,^^42^ and PAT^12^^,^^13^ imaging, have been reported.

In biometric authentication using OCT fingerprinting, the field of view was several tens of millimeters squared, with an image acquisition time of 8 s and image processing completed in <1  s.^42^ The OCT imaging time is approximately the same as the hyperspectral imaging time with dual light illumination in the study conducted herein (Sec. S3 in the Supplementary Material). In addition, the EER in this previous study was 2.7%,^42^ whereas the hyperspectral personal identification method proposed in this study achieved a lower EER. Furthermore, owing to the impact of skin deformation on OCT measurements, it is not possible to press the finger against a glass plate, which makes it difficult to align the finger position. Conversely, the technique proposed herein allows for the entire palm to be pressed against a glass plate for stable measurements. Using MediaPipe Hands, the ROI is automatically set on the palm. Although the length of ROIs varied with each measurement (Table S1 in the Supplementary Material), the accuracy in terms of personal identification remained high. This suggests that despite changes in the degree of pressure applied to the hand, the annotated positions were relatively consistent, segmenting almost the same location. Even when the palm was tilted and partially lifted from the glass plate, the length of the ROI varied (Table S3 in the Supplementary Material); however, the relative landmark positions remained (Figs. S5, S8, and S10 in the Supplementary Material). Motion artifacts caused image misalignment when the palm was completely lifted (Fig. S10 in the Supplementary Material). However, UMAP clustering results were plotted near the baseline condition cluster (Fig. S11 in the Supplementary Material). This may be caused by the fact that hyperspectral imaging, with its lower spatial resolution compared with OCT, is less affected by movements during voluntary hand stabilization. In another study using OCT images of internal fingerprint structure for anti-spoofing, the processing time is ∼2  s; however, the non-local means denoising process for OCT images requires considerable time (∼370  s).^11^ Conversely, the proposed method used only the 3D Gaussian filter for denoising, which required <1s. The EER in the OCT study was 3.57%. However, the results of the study conducted herein demonstrate a lower EER (0.04%), indicating that the proposed hyperspectral method achieves higher accuracy.

Biometric authentication using 3D finger vein structures obtained from PAT requires 35 s for imaging.^12^ In addition, the imaging process requires the finger to be in contact with a water tank with the use of ultrasound gel. The EER in this PAT study was 0.13%, whereas the proposed method achieved a lower EER. Another biometric identification study using PAT of fingerprints and underlying vasculature requires 60 s for imaging.^13^ This PAT approach also requires contact with a water tank, albeit without the necessity for ultrasound gel. Both PAT studies had longer image acquisition times compared with the proposed method. Also, in the first PAT study, the evaluation of rotation invariance was conducted by rotating the finger 30 deg clockwise and counterclockwise during imaging. The results showed that such rotation caused body motion and poor contact, which affected the authentication process. This study also investigated the hand tilt effect. The results showed that as the tilt angle increased, the plots in the UMAP embedding space tended to spread out (Fig. S6 in the Supplementary Material). However, they remained clustered at a distance from those of other individuals. Therefore, the proposed method is considered to have high rotation invariance. Furthermore, the effect of light source position was investigated. As with the tilt effect, the results showed that similar tendencies occurred in the UMAP embedding space (Fig. S9 in the Supplementary Material).

The authentication process should be as fast as possible to ensure a positive user experience. Operational requirements define a 10-s maximum period for practical applications.^42^^,^^43^ In 4-band multispectral palm imaging, the imaging time is <1  s.^44^ The image acquisition time for palm hyperspectral imaging with 321 wavelengths takes 10.7 s,^18^ which is shorter than that for my hyperspectral imaging with 121 wavelengths. However, in this study, the image acquisition time took 24 s (20  lines/s). In addition, image processing time took ∼6.6  s. Although this lengthier imaging time frame may present a limitation for practical applicability, it should be noted that the primary purpose of this study was to demonstrate the potential for personal identification using cross-sectional hyperspectral images. Therefore, priority was afforded to image quality herein. However, future research directions could concentrate on improving the practical applicability of the proposed solution in terms of the time required for image acquisition/processing. The burden on the subjects was also considered, such as thermal and visual stimulations caused by increasing the light intensity. However, for one subject, an additional imaging experiment was conducted by increasing the light intensity using dual light sources and decreasing the exposure time (Sec. S3 in the Supplementary Material). This demonstrated that a palm hyperspectral image, which can be used for personal identification, can be acquired in 8 s (60  lines/s). Therefore, it was confirmed that imaging time could be reduced. Furthermore, MediaPipe Hands was used to set the ROI, and it was necessary to scan the entire hand. With an image recognition library focused on a narrower palm region, the scanning area could be reduced, which in turn could further reduce imaging time. In addition, the average ROI scan-line number was 180 lines. Therefore, the imaging time could be further reduced to 3 s (180/60  lines/s) by first determining the ROI and subsequently scanning only lines within that ROI. In this study, all image processing was performed using the CPU. In addition, the processing time is expected to be significantly reduced using general-purpose graphics processing units. These optimizations suggest that image processing using the proposed method could be realistically completed within 10 s.

A hyperspectral imaging system primarily consists of a hyperspectral camera and light source. Therefore, camera and light source characteristics, such as spectral response, exposure time, light intensity, and spectral distribution, are expected to affect the imaging result. In additional experiments as detailed in Sec. S3 in the Supplementary Material, the potential for hyperspectral personal identification was demonstrated by increasing light source intensity while reducing exposure time. In this study, spectral distribution was corrected using the white balance for each experiment. However, it is difficult to detect characteristic peaks from absorbers in the body when using light sources with significantly different spectral distributions.^45^ This could affect the patterns in hyperspectral cross-sectional images and potentially hinder personal identification. Moreover, the spectral response was not evaluated herein because the experiments were conducted using a single hyperspectral camera. The camera used in this study operated in the visible to near-infrared range (400 to 1000 nm). A previous study presented spectral data of skin in the 1000- to 2500-nm range, which significantly differed from the visible to near-infrared spectrum.^46^ This indicates potential differences in spectral response that could affect imaging results. However, it was also reported that the spectral data of skin in the 1000- to 2500-nm range showed less inter-individual variability compared with the visible to near-infrared spectrum. Therefore, spectral characteristics in the range may be less suitable for personal identification.

This study did not fully address the stability of cross-sectional hyperspectral features over time. The maximum measurement interval was one day. Previous work has demonstrated the stability of hyperspectral measurements by documenting a facial database in the 400- to 1000-nm range over several weeks.^47^ In addition, in biometrics, near-infrared hyperspectral face imaging (700 to 1000 nm) has shown that skin spectral curves offer high long-term stability, performance, uniqueness, and acceptability.^48^ However, palm injuries and health conditions may impact the spectrum, potentially affecting the accuracy of biometric authentication.

In the realm of biometric authentication, the limited size of datasets poses a significant challenge for current research. This constraint impedes the accurate validation of proposed methods. Although this study demonstrates the ability to discriminate between subjects using hyperspectral imaging of the hand palm, the dataset utilized was small-scale, comprising only 10 subjects. To address this data scarcity issue, future work will involve acquiring data from a larger number of subjects to design a more robust authentication system. In addition, hyperspectral imaging devices remain extremely expensive, which limits their widespread adoption in various solutions. However, hyperspectral imaging for human skin has been applied in clinical settings. Integrating the proposed method into these clinical applications may enhance their usability. Therefore, it is important to note that personal identification using hyperspectral imaging offers several advantages that standard solutions have yet to achieve.

## 5 Conclusion

This paper proposes biometric authentication using hyperspectral images of the palm, ranging from visible to near-infrared wavelengths with 5-nm resolution. First, a machine learning-based method for ROI detection is introduced. Subsequently, feature vectors are extracted from cross-sectional hyperspectral images in the sagittal direction, capturing spectral connectives and skin surface morphology. Finally, the effectiveness of the proposed method for biometric authentication is evaluated using dimension reduction techniques. The evaluation results indicate that hyperspectral personal identification can achieve good performance. This suggests that cross-sectional hyperspectral imaging has the capability to differentiate between subjects, potentially paving the way for innovative, secure, and efficient identification methods.

## Supplementary Material



## Data Availability

The data are not publicly available because of privacy concerns related to the research participants. In addition, the code is not publicly available because of restrictions.
