# Supplementary material for: Personal identification using a cross-sectional hyperspectral image of a hand
Source: J Biomed Opt. 2024 Dec 16;30(2):023514. doi: 10.1117/1.JBO.30.2.023514 (PMC11649094; doi:10.1117/1.JBO.30.2.023514)
Supplement: Supplementary file 1 [file JBO_030_023514_SD001.doc]

Supplementary Material

Personal identification using Cross-sectional hyperspectral image of hand

Takashi Suzuki

1. **Hyperspectral image of palm**

The lengths of the region of interest (ROI) set on palm images using MediaPipe Hands are shown in Table S1.

**Table S 1** Lengths of line ROIs on palm images.

| **Line ROI lengths (mm)** | | | | | | | | | | |
| --- | --- | --- | --- | --- | --- | --- | --- | --- | --- | --- |
| Measurement | Sub.1 | Sub.2 | Sub.3 | Sub.4 | Sub.5 | Sub.6 | Sub.7 | Sub.8 | Sub.9 | Sub.10 |
| 1 | 69.30 | 70.50 | 70.79 | 74.05 | 72.05 | 69.33 | 85.45 | 85.51 | 68.67 | 76.93 |
| 2 | 67.45 | 69.31 | 70.55 | 76.90 | 73.47 | 75.71 | 85.53 | 88.27 | 69.18 | 75.85 |
| 3 | 73.93 | 75.60 | 71.42 | 77.70 | 79.23 | 70.76 | 84.72 | 82.47 | 69.04 | 75.40 |
| 4 | 74.35 | 69.79 | 66.37 | 68.49 | 76.71 | 74.19 | 86.76 | 90.00 | 70.86 | 77.43 |
| 5 | 64.74 | 72.99 | 66.41 | 72.24 | 76.17 | 74.97 | 83.36 | 89.28 | 69.15 | 78.03 |
| 6 | 77.35 | 73.57 | 70.72 | 78.54 | 71.33 | 74.65 | 84.62 | 87.63 | 69.88 | 76.69 |
| 7 | 79.81 | 69.84 | 72.26 | 80.84 | 76.76 | 79.85 | 87.18 | 88.52 | 69.26 | 78.18 |
| 8 | 77.29 | 74.10 | 69.26 | 73.96 | 68.63 | 82.54 | 85.12 | 90.33 | 68.70 | 76.33 |
| 9 | 81.16 | 70.50 | 72.32 | 69.73 | 76.00 | 81.32 | 87.79 | 86.40 | 70.39 | 78.60 |
| 10 | 83.16 | 68.53 | 69.54 | 72.28 | 79.93 | 80.11 | 85.95 | 85.77 | 70.39 | 77.84 |
| **Mean** | 74.89 | 71.47 | 69.96 | 74.47 | 75.03 | 76.34 | 85.65 | 87.42 | 69.55 | 77.13 |
| **SD** | 6.14 | 2.39 | 2.12 | 3.97 | 3.58 | 4.47 | 1.32 | 2.41 | 0.77 | 1.06 |

**
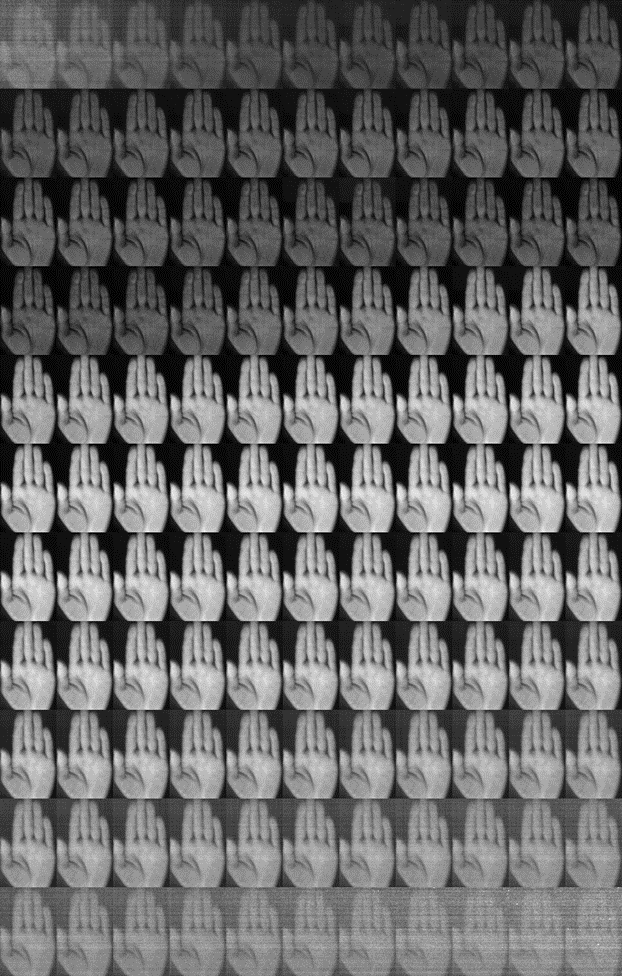
**

Fig. S1 An example of hyperspectral hand images from Subject 1. From left to right, top to bottom, wavelength is increased from 400–1000 nm with 5 nm intervals. Each image is 280 × 440 px in size, with dimensions of 100.8 ×184.8 mm.

**2. Feature histograms**


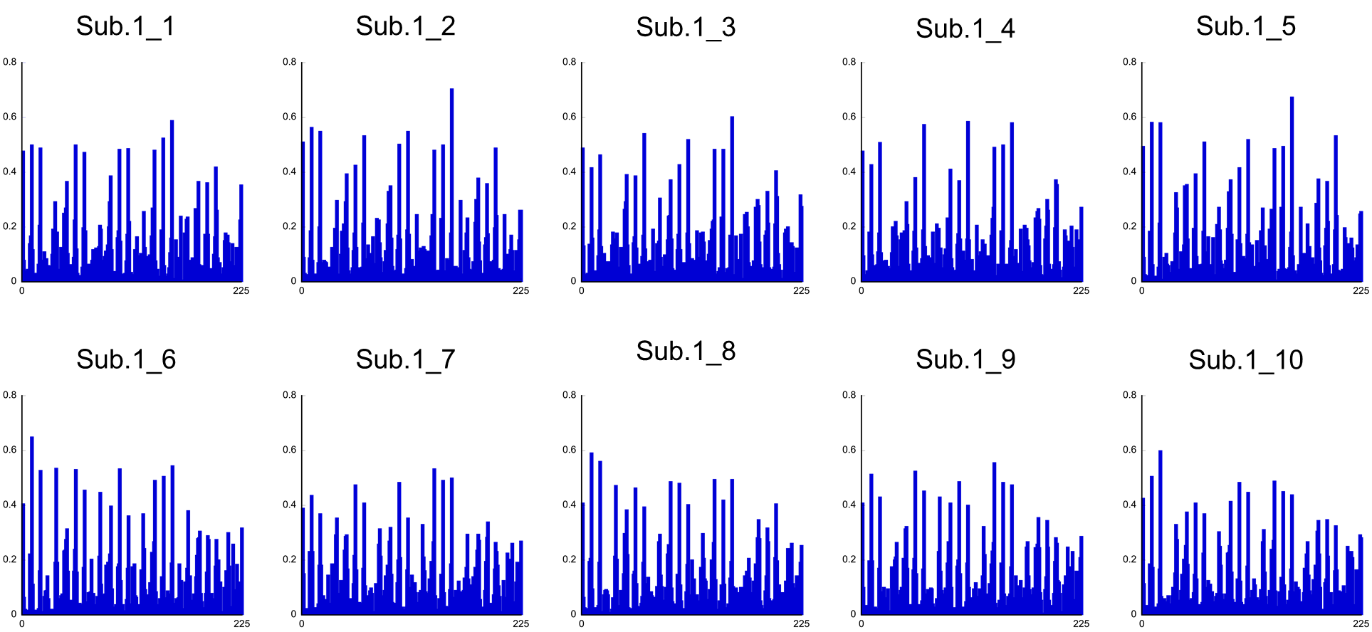


Fig. S2 Subject 1 feature histograms.


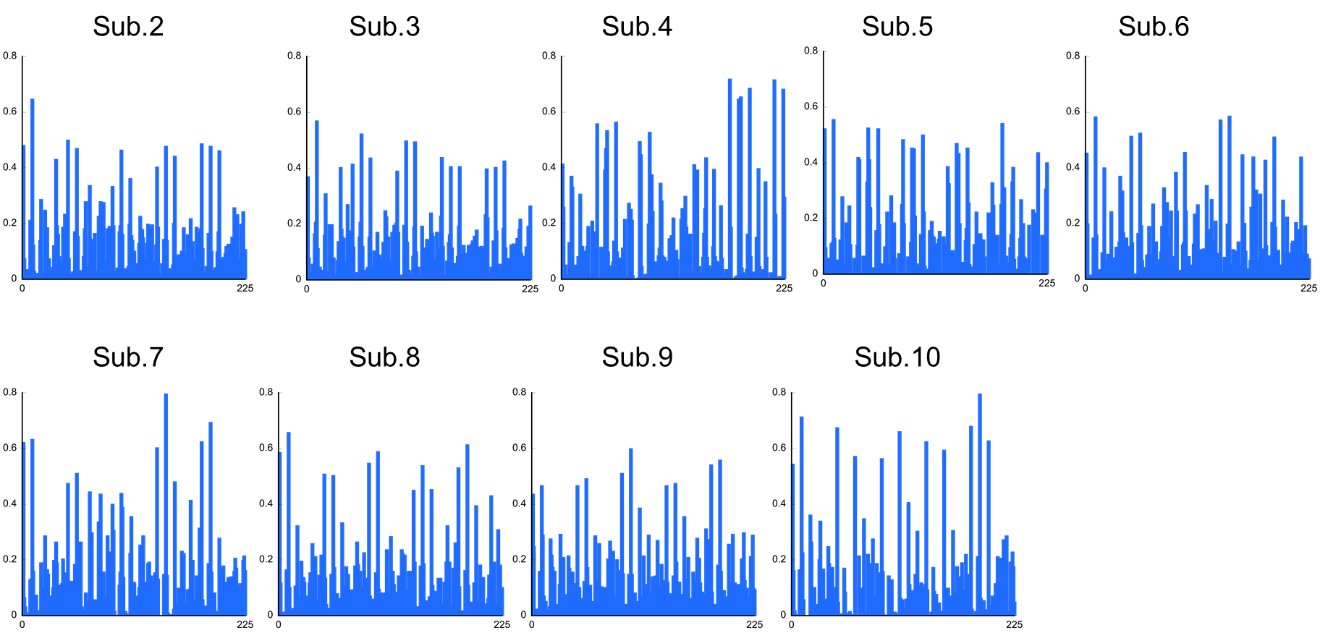


Fig. S3 Feature histograms from Subjects 2–10.

# 3. Evaluation of effects of various palm imaging conditions on personal identification

To examine the effects of hand and light source position and light intensity on feature extraction, additional imaging experiments were conducted under some conditions that differed from the main experiment as follows. For this experiment, the data was acquired from a single subject (Subject A), a 47 year old healthy adult male. The hyperspectral imaging method, regions of interest (ROI) setting, and feature extraction were identical to that of the main experiment. For clustering, uniform manifold approximation and projection (UMAP) was used, which showed the highest accuracy in the main experiment. Specific imaging conditions were as follows.

*3.1 Imaging conditions*

*3.1.1 Effect of palm tilt angle*

In addition to the hand being in contact with the glass, it was tilted approximately 5, 10, 20, and 30 degrees in both the lateral and forward/backward directions, with five images taken under each condition. The tilt directions are shown in Fig. S4.

To monitor the hand tilt angle, the standard iPhone app Level was used. The device used was an iPhone 13 mini running iOS version 17.6.1. The app was launched, and the iPhone was positioned on the back of the hand with the palm resting on a glass surface. After zeroing the level, the hand was tilted to the desired angle and held still for the measurement.

For the palm hover and supported hover conditions, the palm was lifted approximately 10 mm from the pressed position and held in that position for the scan period.


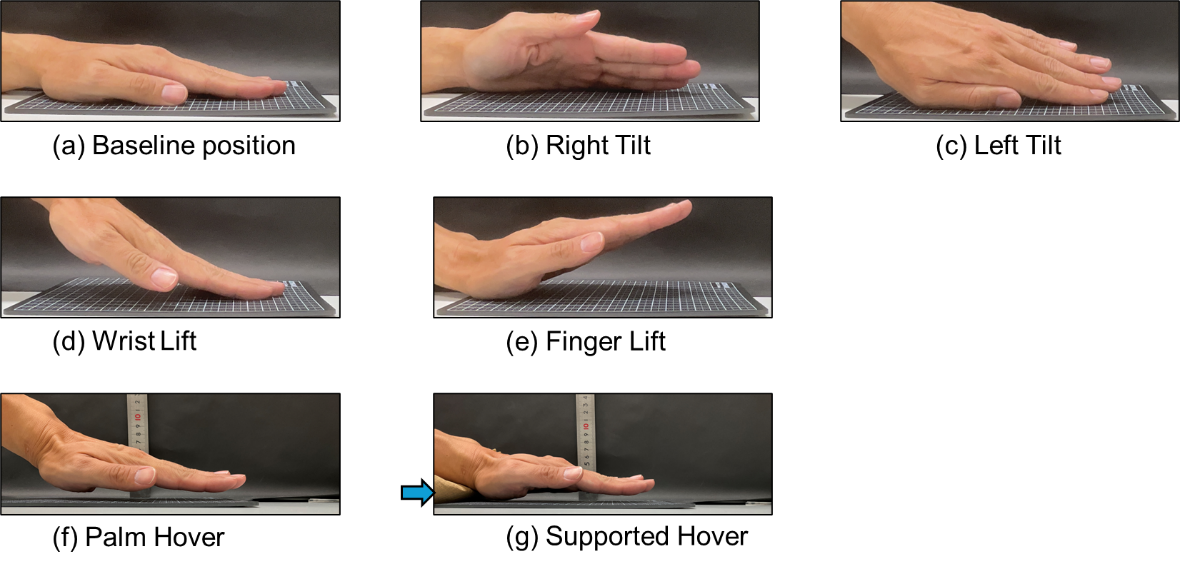


**Fig. S4** Illustration of various palm tilting for palm imaging. Except for baseline position (a), imaging was performed with the palm tilted approximately 5, 10, 20, and 30 degrees for each tilt condition (b-g), with five times for each angle. For the supported hover condition, a rolled paper wiper which is indicated by the blue arrow in the Fig. 4(g) was placed under the wrist to act as a palm rest to support the wrist during imaging.


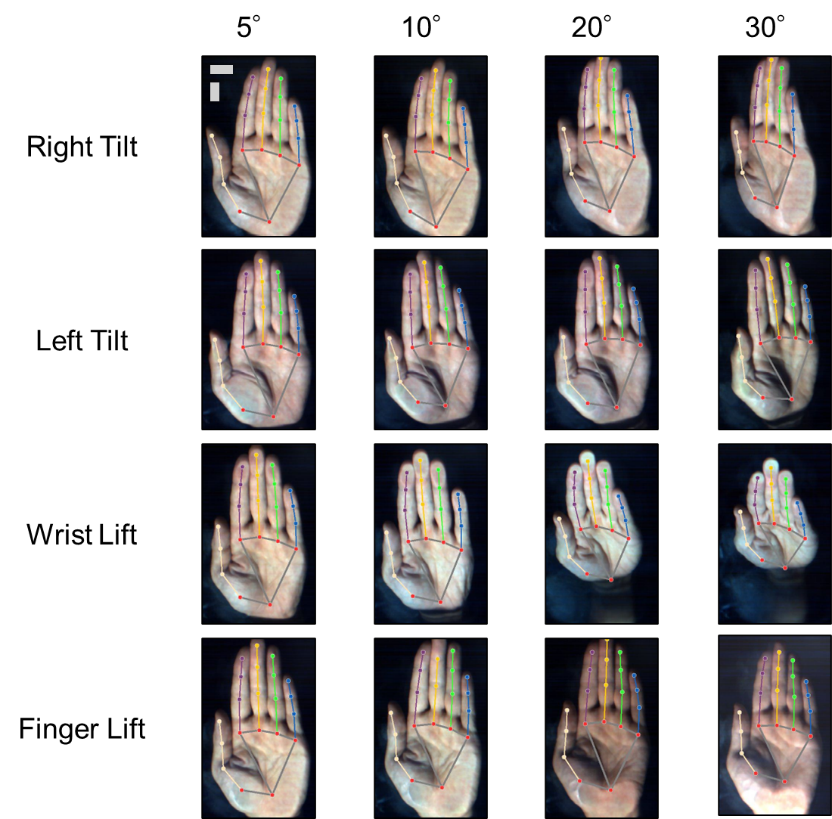


**Fig. S5** Palm images captured at various angles with landmarks overlaid using MediaPipe Hands. Imaging experiments were conducted on an additional subject (Subject A). Rows represent imaging conditions, while columns represent tilt angles. The horizontal and vertical scale bars in the top-left image indicate 20 mm.

For each palm tilt condition, Subject A data from the additional experiment and Subjects 4, 7, and 10 data (taken in the baseline position) from the main experiment, which were randomly selected, were clustered using UMAP.


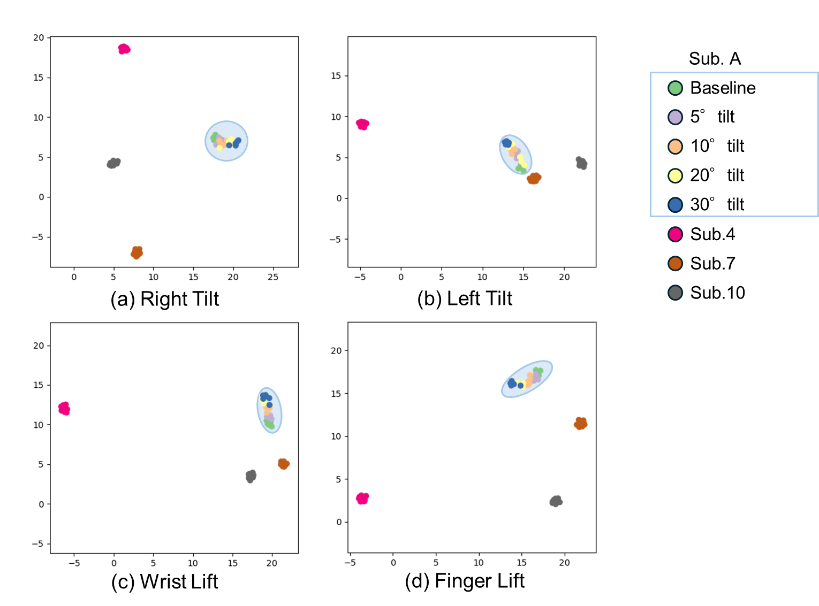


**Fig. S6** UMAP clustering under various tilt conditions. Each graph shows a different tilt condition from the additional subject (Subject A) and Subjects 4, 7, and 10 from the main experiment. The five colors (green, purple, orange, yellow, and blue) represent different tilt conditions for Subject A. The remaining colors represent data for each subject. Although the data from Subject A were clustered, variability owing to different tilt angles was observed. Subject A data are enclosed in a light blue frame in the graph.

*3.2 Effect of light source position and light intensity*

The positions of illuminating light sources for each condition are shown in Fig. S7. Under the dual illumination conditions, the scan rate was set at 60 lines/s, and camera exposure time was set at 0.017 s (17 ms). The total scan time was 8 s. In addition, the glass surface temperature increased to approximately 3 °C in 10 s and approximately 5 °C in 30 s.


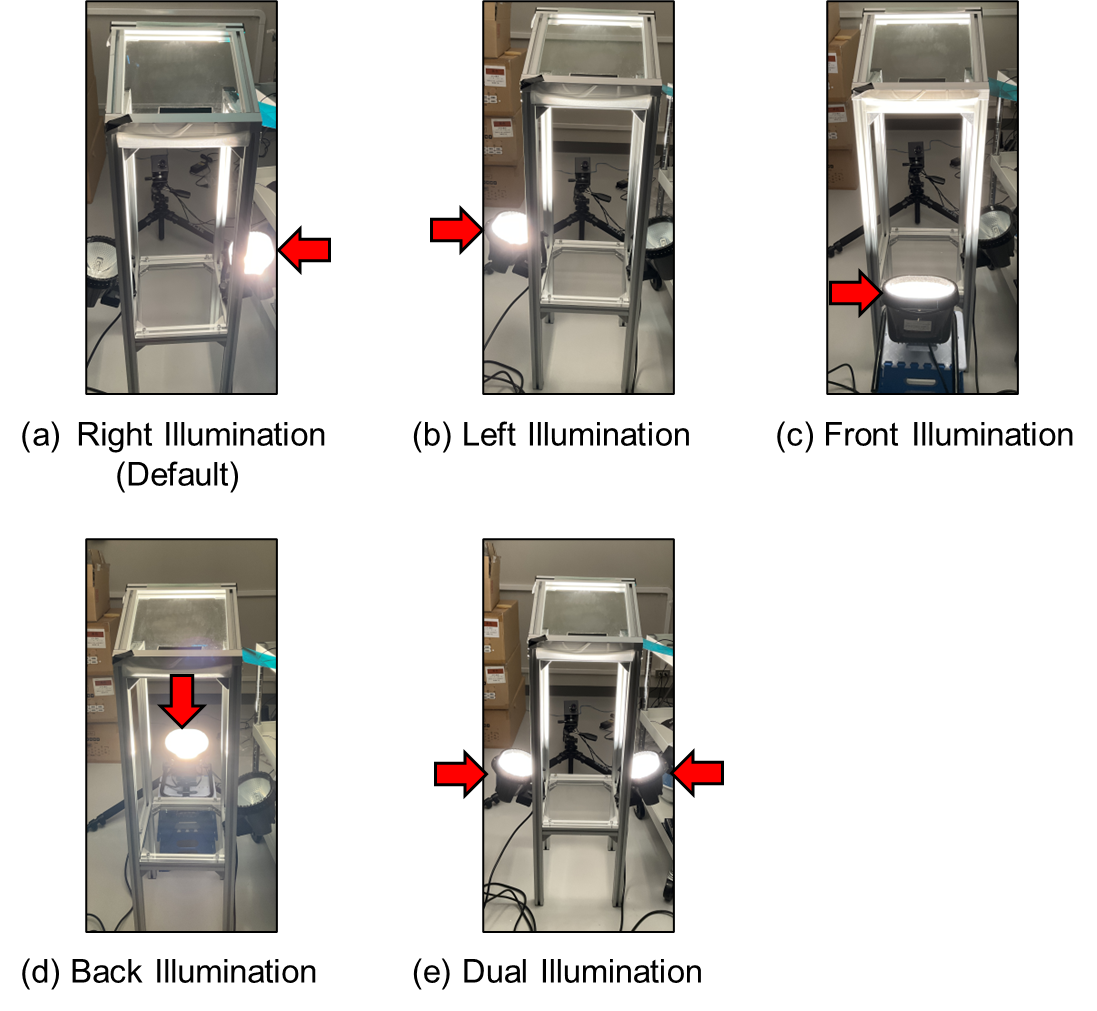


**Fig. S7** Illumination conditions for investigating lighting effects. Red arrows indicate light sources currently switched on.


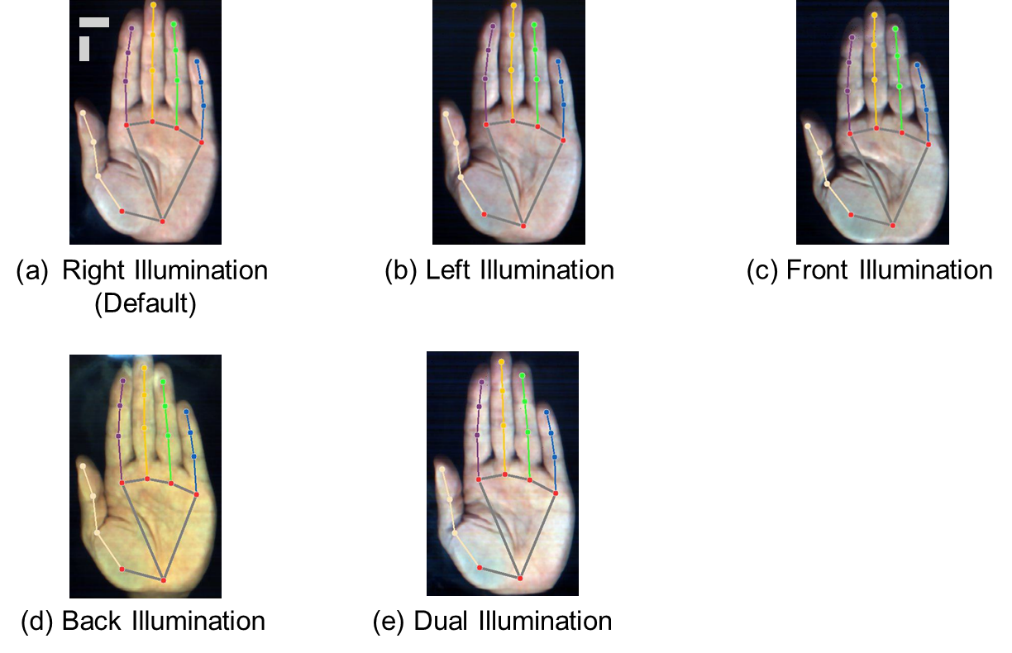


**Fig. S8** Palm images captured under different lighting conditions with landmarks overlaid using MediaPipe Hands. Imaging experiments were conducted on an additional subject (Subject A). The horizontal and vertical scale bars in the top-left image indicate 20 mm.

The results for Subject A at four different light source positions and using dual light sources were clustered and plotted using UMAP, along with Subject 4, 7, and 10 data.


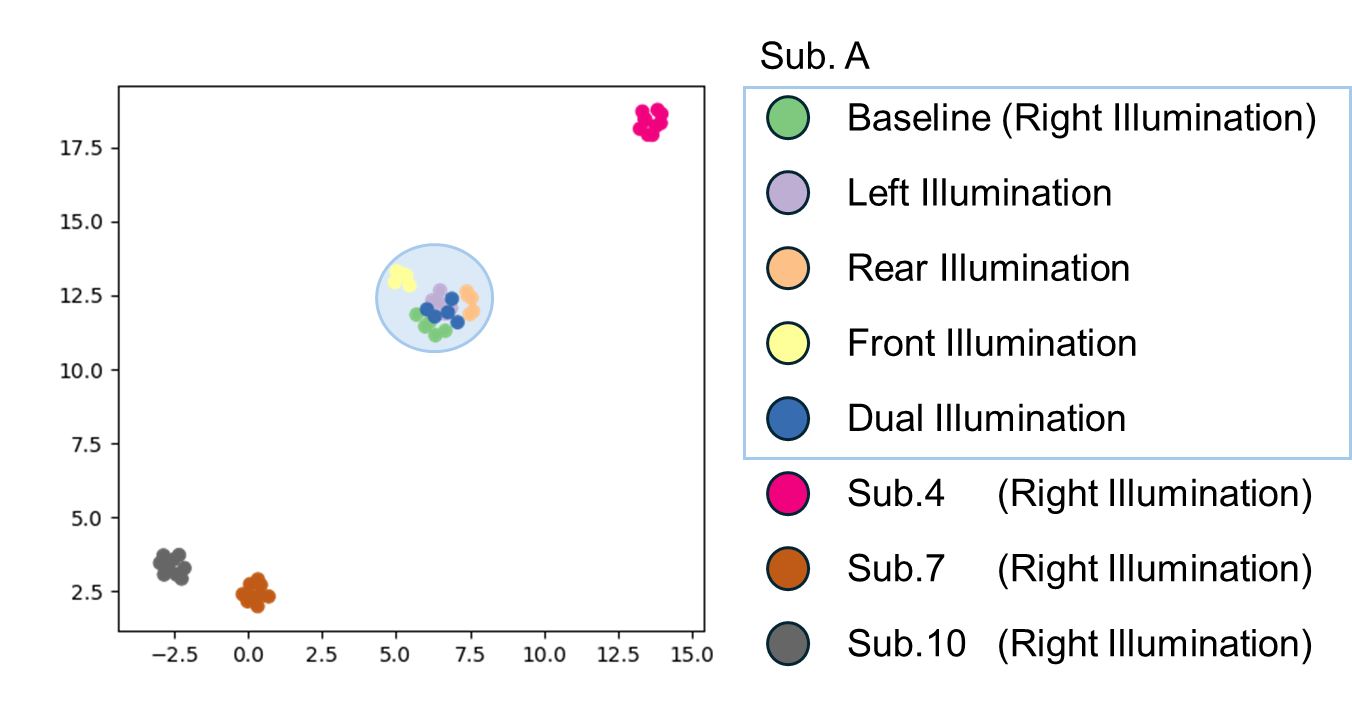


**Fig. S9** UMAP clustering under different lighting conditions. The graph shows a different lighting condition from the additional subject (Subject A) and Subjects 4, 7, and 10 from the main experiment with baseline conditions. The five colors (green, purple, orange, yellow, and blue) represent different tilt conditions for Subject A. The remaining colors represent data for each subject. Subject A data are enclosed in a light blue frame in the graph.

*3.3 Effect of motion artifacts*


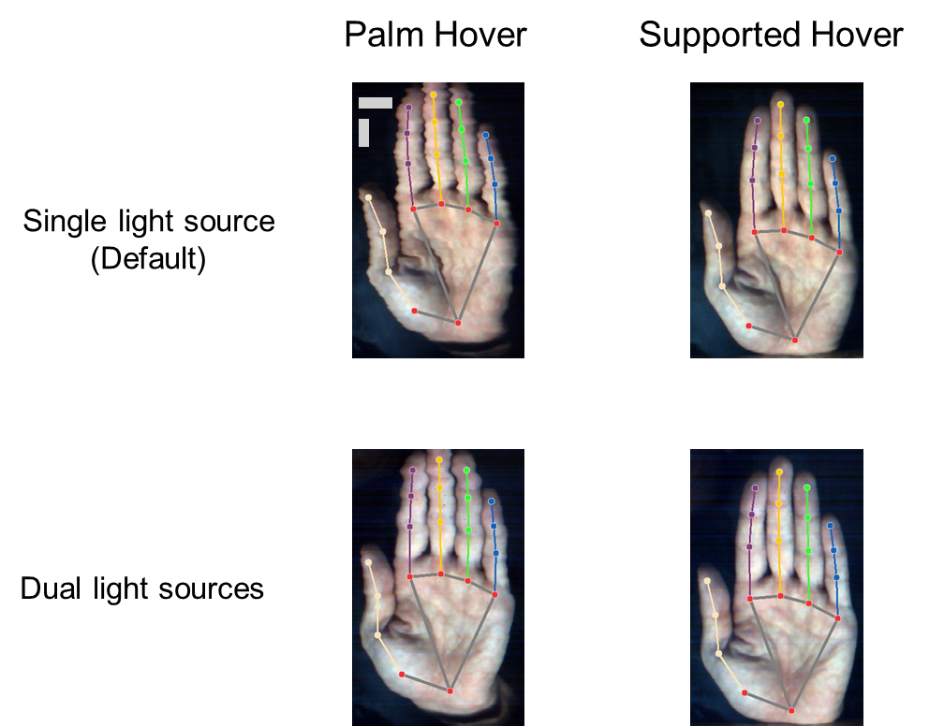


**Fig. S10** Hand images with landmarks overlaid using MediaPipe Hands during palm hovering. The left column shows acquired data with the palm raised. The right column shows images where the palm is hovering, however, the wrist is resting on a palm rest. Imaging with a single light source in the top row required a longer exposure time which resulted in approximately 24 s imaging time, whereas that with dual light sources in the bottom row required a shorter exposure time resulting in approximately 8 s imaging time. The horizontal and vertical scale bars in the top-left image indicate 20 mm.

In addition, Subject A data with or without palm contact with the glass and the data from Subjects 4, 7, and 10 (taken in the baseline position) were also clustered using UMAP.


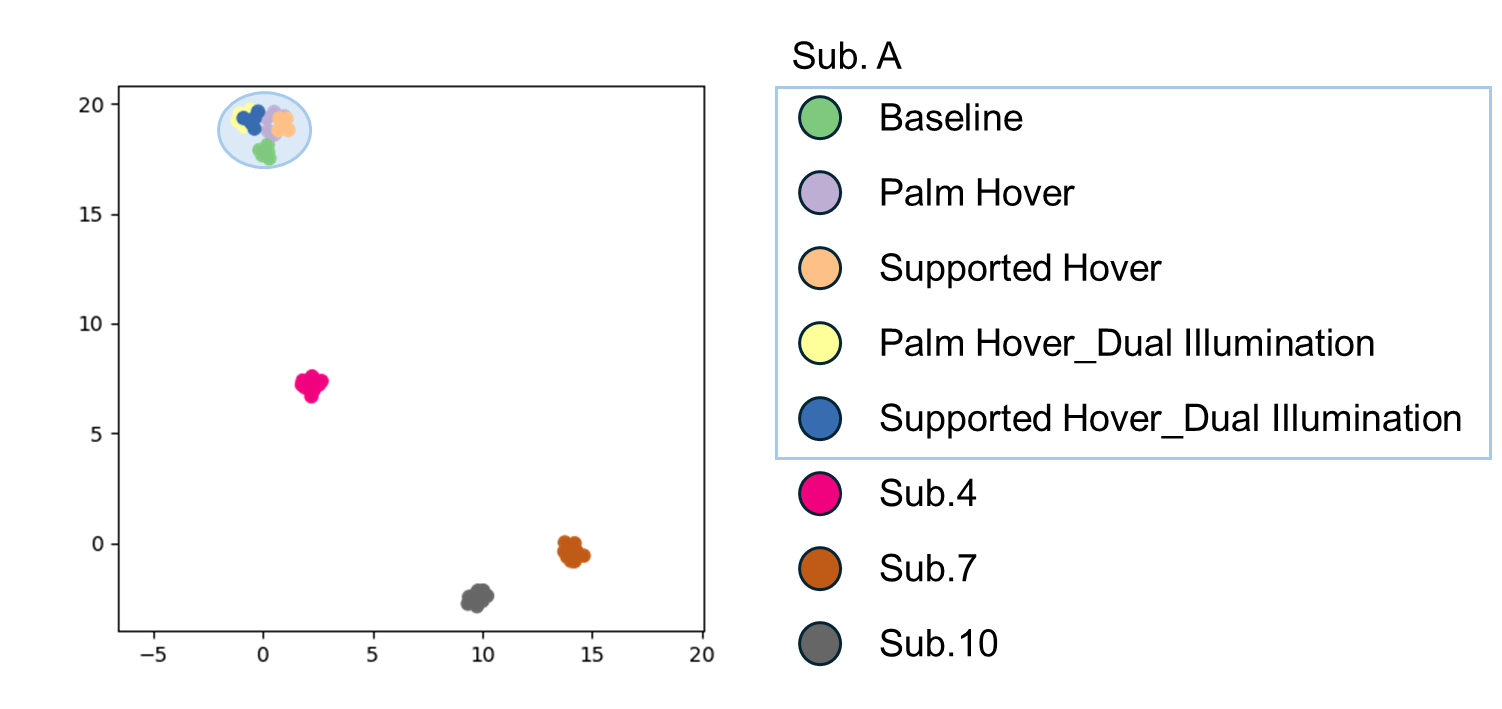


**Fig. S11** UMAP clustering result demonstrates the effect of pressing the palm on the glass or not. The graph shows a different tilt condition from the additional subject (Subject A) and Subjects 4, 7, and 10 from the main experiment. The five colors (green, purple, orange, yellow, and blue) represent different tilt conditions for Subject A. The remaining colors represent data for each subject. Subject A data are enclosed in a light blue frame in the graph.

**4. Clustering quality evaluation based on UMAP**

To evaluate the level of cohesion on the 2D UMAP plot for each condition, the average intra-cluster distance, intra-cluster cohesion, and inter-cluster distance were employed. The results of this evaluation are presented in Table S2. The average intra-cluster distance was calculated as the average distance between all pairs of data points within a cluster. Intra-cluster cohesion was calculated as the sum of the distances from each point to the centroid of a cluster. Moreover, the inter-cluster distance was calculated as the distance between the centroids of the clusters. In the intra-cluster evaluations, the data from Subject A’s baseline position condition (in the first row in Table S2) was treated as a single cluster. All other items were evaluated as an intra-cluster, with the value of the baseline position condition and each condition (e.g., Baseline position values and Right Tilt_5 degree values) treated as a single cluster. Also, the condition “Sub. A” indicated that the data for all conditions of Subject A were treated as a single cluster. The inter-cluster evaluation was performed only on Subject A, comparing the clusters between the baseline position condition and each of the measurement conditions. In all evaluations, smaller values indicated a higher level of cohesion. All data were projected onto a single UMAP space. These results are presented in Table S3. Noteworthy is that, the average values of the average intra-cluster distance and intra-cluster cohesion for Subjects 1–10 in the main experiment were 0.24 ± 0.08 and 1.64 ± 0.60, respectively.

**Table S2** Cluster variability evaluation

| **Condition** | **Average intra-cluster distance** | **Intra-cluster cohesion** | **Inter-cluster distance** |
| --- | --- | --- | --- |
| **Baseline Position** | 0.41 | 1.24 |  |
| **Right Tilt_5 degrees** | 0.40 | 2.74 | 0.16 |
| **Right Tilt_10 degrees** | 0.58 | 3.80 | 0.04 |
| **Right Tilt_20 degrees** | 1.24 | 9.50 | 0.69 |
| **Right Tilt 30 degrees** | 1.46 | 11.53 | 1.00 |
| **Left Tilt_5 degrees** | 2.05 | 15.87 | 1.58 |
| **Left Tilt_10 degrees** | 2.27 | 18.82 | 1.87 |
| **Left Tilt_20 degrees** | 2.56 | 19.01 | 1.75 |
| **Left Tilt_30 degrees** | 2.37 | 20.26 | 2.02 |
| **Wrist Lift_5 degrees** | 0.79 | 6.06 | 1.00 |
| **Wrist Lift_10 degrees** | 2.62 | 21.75 | 1.81 |
| **Wrist Lift_20 degrees** | 2.97 | 24.68 | 2.45 |
| **Wrist Lift_30 degrees** | 2.97 | 24.90 | 2.48 |
| **Finger Lift_5 degrees** | 0.57 | 3.73 | 0.23 |
| **Finger Lift_10 degrees** | 1.46 | 11.50 | 1.13 |
| **Finger Lift_20 degrees** | 2.51 | 19.45 | 1.93 |
| **Finger Lift_30 degrees** | 3.01 | 25.40 | 2.52 |
| **Palm Hover** | 0.85 | 6.45 | 0.62 |
| **Supported Hover** | 0.89 | 6.65 | 0.63 |
| **Left Illumination** | 0.55 | 3.87 | 0.33 |
| **Front Illumination** | 1.07 | 8.74 | 0.87 |
| **Back Illumination** | 1.00 | 7.56 | 0.73 |
| **Dual Illumination** | 0.6 | 4.08 | 0.37 |
| **Palm Hover**  **Dual Illumination** | 1.11 | 8.5864 | 0.83 |
| **Supported Hover**  **Dual Illumination** | 1.21 | 9.76 | 0.96 |
| **Sub. A** | 2.46 | 223.80 |  |

**Table S3** Averaged segmentation distances of line ROIs in additional experiments

| **Condition** | **Average segmentation distances**  **(mm)** | **Standard Deviation**  **(mm)** |
| --- | --- | --- |
| **Baseline Position** | 79.64 | 2.53 |
| **Right Tilt_5 degrees** | 78.75 | 1.24 |
| **Right Tilt_10 degrees** | 80.72 | 2.08 |
| **Right Tilt_20 degrees** | 75.78 | 4.04 |
| **Right Tilt 30 degrees** | 71.16 | 2.64 |
| **Left Tilt_5 degrees** | 80.80 | 1.71 |
| **Left Tilt_10 degrees** | 75.09 | 6.30 |
| **Left Tilt_20 degrees** | 70.49 | 8.27 |
| **Left Tilt_30 degrees** | 64.32 | 4.84 |
| **Wrist Lift_5 degrees** | 73.06 | 3.43 |
| **Wrist Lift_10 degrees** | 66.62 | 2.27 |
| **Wrist Lift_20 degrees** | 60.27 | 1.56 |
| **Wrist Lift_30 degrees** | 51.59 | 4.46 |
| **Finger Lift_5 degrees** | 69.87 | 2.71 |
| **Finger Lift_10 degrees** | 65.53 | 4.22 |
| **Finger Lift_20 degrees** | 68.64 | 6.58 |
| **Finger Lift_30 degrees** | 59.24 | 5.49 |
| **Palm Hover** | 76.38 | 5.70 |
| **Supported Hover** | 77.52 | 1.11 |
| **Left Illumination** | 81.23 | 2.82 |
| **Front Illumination** | 76.91 | 0.93 |
| **Back Illumination** | 82.18 | 0.41 |
| **Dual Illumination** | 82.02 | 1.82 |
| **Palm Hover**  **Dual Illumination** | 80.52 | 2.33 |
| **Supported Hover**  **Dual Illumination** | 78.34 | 1.97 |
